# Molecular epidemiology of Marek’s disease virus in central Pennsylvania, USA

**DOI:** 10.1093/ve/vey042

**Published:** 2019-04-23

**Authors:** Andrew S Bell, David A Kennedy, Matthew J Jones, Christopher L Cairns, Utsav Pandey, Patricia A Dunn, Moriah L Szpara, Andrew F Read

**Affiliations:** 1 Department of Biology, The Pennsylvania State University, University Park, PA, USA; 2 Department of Entomology, The Pennsylvania State University, University Park, PA, USA; 3 Center for Infectious Disease Dynamics, The Pennsylvania State University, University Park, PA, USA; 4 Department of Biochemistry and Molecular Biology, The Pennsylvania State University, University Park, PA, USA; 5 Animal Diagnostic Laboratory, Department of Veterinary and Biomedical Sciences, The Pennsylvania State University, University Park, PA, USA

**Keywords:** Marek's disease virus, haplotype diversity, pathogen persistence, virulence, vaccine resistance

## Abstract

The evolution of Marek’s disease virus (MDV, *Gallid herpesvirus 2*) has threatened the sustainability of poultry farming in the past and its continued evolution remains a concern. Genetic diversity is key to understanding evolution, yet little is known about the diversity of MDV in the poultry industry. Here, we investigate the diversity of MDV on 19 Pennsylvanian poultry farms over a 3-year period. Using eight polymorphic markers, we found that at least twelve MDV haplotypes were co-circulating within a radius of 40 km. MDV diversity showed no obvious spatial clustering nor any apparent clustering by bird line: all of the virus haplotypes identified on the commercial farms could be found within a single, commonly reared bird line. On some farms, a single virus haplotype dominated for an extended period of time, while on other farms the observed haplotypes changed over time. In some instances, multiple haplotypes were found simultaneously on a farm, and even within a single dust sample. On one farm, co-occurring haplotypes clustered into phylogenetically distinct clades, putatively assigned as high and low virulence pathotypes. Although the vast majority of our samples came from commercial poultry farms, we found the most haplotype diversity on a noncommercial backyard farm experiencing an outbreak of clinical Marek’s disease. Future work to explore the evolutionary potential of MDV might therefore direct efforts toward farms that harbor multiple virus haplotypes, including both backyard farms and farms experiencing clinical Marek’s disease.

## 1. Introduction

Marek’s disease virus (MDV) is a highly contagious oncogenic herpesvirus that costs the global poultry industry more than $US1 billion annually (Morrow and Fehler 2004). Over the second half of last century, MDV evolved in two notable ways. First, viral evolution eroded the efficacy of initially very protective vaccines (Witter 1997). MDV is one of the few well-documented case studies of the evolution of vaccine resistance (Kennedy and Read 2017, 2018). Second, MDV virulence increased dramatically (Witter 1997; Osterrieder et al. 2006). When the disease was first described, it was characterized by transient paralysis in older birds; today, strains circulate which kill all unprotected birds in less than 10 days (Read et al. 2015). If these evolutionary trajectories were to continue, the economic burden of the disease could substantially increase. Here we describe the molecular epidemiology of currently circulating MDV haplotypes to gain insight into the evolutionary potential of this economically costly and intellectually intriguing pathogen. 

MDV, also known as *Gallid herpesvirus 2* (GaHV-2), belongs to the genus *Mardivirus* of the subfamily *Alphaherpesvirinae*. The virus was originally believed to exist as three serotypes designated as MDV-1, MDV-2, and MDV-3. We now know that MDV-2 (or *Gallid herpesvirus 3*) and MDV-3 (*Meleagrid herpesvirus 1* or herpesvirus of turkeys (HVT)) are distinct viral species that do not cause Marek’s disease (note that for this reason we reserve the term ‘MDV’ for use in reference to GaHV-2). Strains of HVT, GaHV-3, and an attenuated strain of GaHV-2 called the Rispens vaccine strain comprise three generations of vaccines that are used to control Marek’s disease in chickens. None of these vaccines fully curtail infection and transmission of wild-type virus, meaning that wild-type MDV can potentially circulate indefinitely on vaccinated farms.

The molecular epidemiology of MDV is in its infancy. Part of the reason is that very little is known about the dynamics of MDV in the poultry industry, and so suitable spatial and temporal scales for study are unknown. Previous studies have investigated diversity at individual genes (e.g., *meq*, multiple glycoproteins, lytic antigen *pp38*, *vIL-8*), but these studies have typically involved small numbers of isolates collected over large geographic regions (Shamblin et al. 2004; Tian et al. 2011; Renz et al. 2012; Wajid et al. 2013). If MDV populations harbor spatial diversity at fine scales or temporal diversity within a single spatial location, these previous studies may provide misleading descriptions of MDV diversity.

To our knowledge, only one prior study has looked in depth at the diversity of MDV present in a particular chicken farm at a particular time (Pandey et al. 2016). The samples used in that whole-genome study contained only a single MDV genotype, but it is unknown whether that lack of diversity is typical. Moreover, we are unaware of any studies that identified and followed viral strains through time—a task that is minimally necessary to describe the evolution of the virus. Recent work has identified genetic markers that appear to correlate with virulence (Padhi and Parcells 2016), and determining whether polymorphisms currently exist at such genetic markers might be particularly useful for studying virulence evolution in real time.

Recently, we conducted a multiyear surveillance study in central Pennsylvania to quantify the spatial and temporal epidemiology of MDV in the poultry industry (Kennedy et al. 2017). This project involved sampling poultry dust from 104 farms over a 3-year period, with five of these farms sampled at weekly intervals. In total, we detected wild-type MDV on 36 of 104 farms. On some farms, MDV was continuously detectable across successive cohorts of birds, often at high levels, while on other farms MDV outbreaks varied in size and duration, and frequently dropped below detectable levels (Kennedy et al. 2017). Variation between farms in rearing practices may contribute to differences in virus dynamics across farms. MDV presence is highly variable between poultry companies (Kennedy et al. 2017), which can be at least partially explained by differences in average cohort duration (Kennedy, Dunn, and Read 2018). Additional differences may arise due to differences in cleanout practices between farms and within farms over time. Cleanout practices can be highly variable, sometimes involving the complete removal of bedding material followed by chemical disinfection and other times involving the reuse of bedding material for multiple flocks.

Here we ask whether viral strains persist on farms over long time scales, or if they instead die out and are replaced by new strains from outside sources. The answer may differ depending on whether virus concentrations drop below detectability. We apply multi-locus genotyping of eight polymorphic markers to a set of virus samples taken from poultry farms in central Pennsylvania. To our knowledge, this study is unique in the depth of its spatial and temporal resolution, in the discriminating power of the genotyping markers, and in using multiple markers to construct and track field haplotypes of MDV. We use these data to determine whether individual farms harbor multiple MDV strains simultaneously and to determine the frequency with which new strains appear on farms. We also use these data to determine whether strain diversity and dynamics differ between different poultry production companies and whether such diversity may depend on the bird strains being reared. Finally, we identify polymorphism at a genetic locus that correlates phylogenetically with virulence, suggesting that multiple pathotypes of MDV may be co-circulating in close geographic range.

## 2. Materials and methods

### 2.1 Overall strategy

Infective MDV develops solely within the feather follicle cells of infected chickens and cell-free virus may be shed with desquamated epithelial cells or, alternatively, in cells associated with feathers lost from these birds (Calnek, Adldinger, and Kahn 1970). Such dust and dander, often termed poultry dust, may remain infective in the environment for weeks or months (Carrozza et al. 1973), with transmission to other chickens occurring upon its inhalation (Colwell and Schmittle 1968; Beasley, Patterson, and McWade 1970). Virus can thus be sampled from birds or poultry dust; in this study, we focused on dust.

We have collected thousands of dust samples as part of a longitudinal study into the prevalence and intensity of Marek’s disease on Pennsylvanian poultry farms (Kennedy et al. 2017). These dust samples had already been screened by PCR for the presence of ‘wild-type’ MDV (defined here as naturally circulating, non-Rispens strains of MDV-1), using assays which distinguish wild-type MDV from vaccine virus strains including the Rispens vaccine (Baigent, Nair, and Le Galludec 2016). In the analysis we report here, we also included five dust samples collected at a single time point from three houses of a backyard flock whose unvaccinated birds were experiencing Marek’s disease.

We selected samples with greater than 500 MDV genome copies per milligram (mg) of dust, which we deemed sufficiently high for genotyping. Full genome sequences can be obtained from dust samples (Pandey et al. 2016), but to date, this is impractical for large numbers of samples, or when virus concentrations are low. We therefore instead used Sanger sequencing-based genotyping to examine potentially polymorphic regions of the genome.

**Figure 1.**
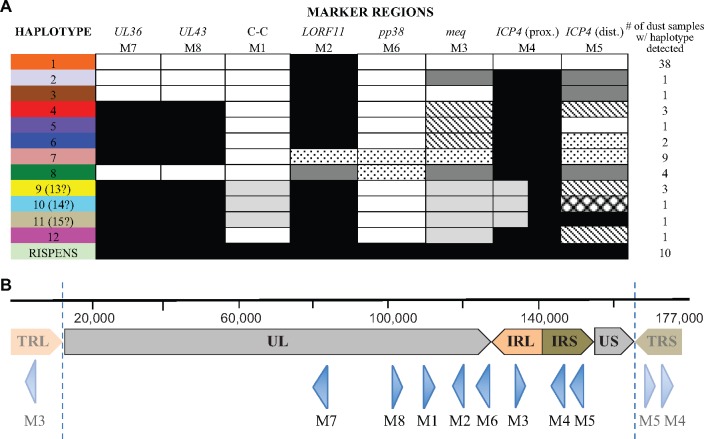
MDV haplotypes present on the thirteen focal Pennsylvanian poultry farms based on eight marker regions and the respective positions of these markers on the MDV genome. (A) Gray-scale/patterned boxes indicate individual variants or alleles for each marker region. Colored bars represent the haplotype collectively described by the eight marker regions. These colors are used subsequently to indicate haplotypes present on individual farms (Figs 2–4). Numbers in the right-hand column indicate the number of dust samples where that haplotype was detected. Cloning of DNA from one particular dust sample revealed two possible genotypes for marker M4 and three possible genotypes for marker M5. Consequently, this may represent as few as three variants (haplotypes 9, 10, and 11: if the gray or black variants of M4 exist exclusively with one of the three possible M5 variants) or as many as six variants (haplotypes 13, 14, and 15: if both M4 variants exist with all 3 possible M5 variants). (B) The scale bar indicates the length of the MDV genome; TRL (terminal repeat long), UL (unique long), IRL (internal repeat long), IRS (internal repeat short), and US (unique short) mark specific regions of the genome; blue triangles (not to scale) represent the position and orientation (3′–5′) of the eight markers; and the dotted vertical lines represent the limits of the terminal repeat regions where certain markers appear a second time within the genome.

Potentially polymorphic regions of the genome were identified as follows. At the onset of the study, ten MDV full genome sequences were available in GenBank. All ten genomes were obtained from cultured isolates and included two attenuated vaccine strains (Table 1). We aligned the sequences for these genomes and examined small (ca. 700 bp) segments for single-nucleotide polymorphisms (SNPs). We identified five regions, each with a minimum of seven polymorphic sites. We additionally added three gene regions of interest. One was a polymorphism in *pp38* (John Dunn, *pers. comm.*). The other two were regions of *UL36* and *UL43* that we identified as polymorphic in another project (unpublished data). This gave us a total of eight marker (M) regions, which we refer to as M1–8. For each of these regions we found between 2 and 6 unique sequence variants (alleles). The gene identities, locations, putative functions, and sizes of these eight marker regions are detailed in Table 2. Using this panel of markers, we were able to identify at least twelve unique multi-locus haplotypes (Fig. 1).

**Table 1.** GenBank accession numbers for *Gallid herpesvirus 2* full genomes used in identifying regions of maximum polymorphism.

Isolate	Country of origin	Pathotype	Accession no.
CV1988, Rispens	Netherlands	attMDV	DQ530348.1
814	China	attMDV	JF742597.1
LMS	China	vMDV	JQ314003
GX0101	China	vvMDV	JX844666.1
CU-2	USA	mMDV	EU499381.1
GA	USA	vMDV	AF147806.2
RB-1B	USA	vvMDV	EF523390.1
Md5	USA	vvMDV	AF243438
Md11	USA	vvMDV	AY510475
648A-p11	USA	vv+MDV	JQ806361

Pathotypes: att, attenuated; m, moderately virulent; v, virulent; vv, very virulent; vv+, very virulent plus.

**Table 2.** Marker regions used for molecular typing in this study.

Marker region	Location (gene)	Position in genome relative to Md5 (AF243438) reference	Amplicon size	Putative gene function
M1	Partial *UL49.5* and *UL50* (C-C)	112076–112841	765	*UL49.5*: membrane glycoprotein involved with immune evasion (Tischer et al. 2002). *UL50*: dUTPase
M2	*LORF11*	124575–125159	584	Associated with viral replication (Lee et al. 2007)
M3	*RLORF7* (*meq*)	5612–6862	1251	Contributes to MDV oncogenesis by facilitating latency entry/reactivation and by mediating transformation of the target cells (Lupiani et al. 2004)
M4	*ICP4* (prox. region)	144162–145010	849	Immediate-early gene located in the R_S_ regions that is thought to play a role in latency and transformation (Xie, Anderson, and Morgan 1996)
M5	*ICP4* (dist. region)	147700–148181	482	
M6	*pp38*	13841–14665	824	Present in R_L_. Involved in the establishment of cytolytic infection in B lymphocytes and latency of infected T cells (Gimeno et al. 2005)
M7	*UL36*	88233–88634	401	Large tegument protein (Klupp et al. 2002)
M8	*UL43*	101766–102106	340	Putative tegument protein

### 2.2 Initial preparation of dust samples

Dust samples were collected, handled, and processed as detailed in Kennedy et al. (2017). In brief, dust samples were collected in 1.5 ml microtubes and stored at 4°C prior to processing. Replicate 2 mg sub-aliquots were then weighed out from each individual dust sample using a Mettler Toledo balance (cat. no. 97035-620). The viral DNA was extracted using the Qiagen DNeasy Blood and Tissue kit (cat. no. 69506) with modifications to the ‘Animal Tissue’ protocol applied to improve extraction efficacy. Extracted DNA was stored at −80°C.

### 2.3 PCR assays and conditions

All PCR amplification assays were performed with the Qiagen Taq PCR Core Kit (cat. no. 201225). Fifty microliter reaction volumes incorporated 5 μl of 10× CoralLoad PCR buffer (MgCl_2_ at a final concentration of 1.5 mM), dNTPs at 200 nM, 1.25 units of Taq, primers at 200 nM, and 10 μl of DNA template. In addition, bovine serum albumin (BSA) was included at a final concentration of 0.08 mg/ml to minimize the effects of PCR inhibitors potentially present in the DNA samples extracted from chicken dust. All PCR reactions were denatured for 2 min at 95°C, annealed for 30 s at the temperature defined in Table 3, extended at 72°C for the duration defined in Table 3, and had a terminal extension for 10 min at 72°C.

**Table 3.** Primer sequences and specific PCR conditions for each marker region.

Marker region	Amplicon size	Primer sequences: 5′–3′’	Specific PCR cycling conditions		
			Annealing temp. (°C)	Extension duration (min)	No. PCR cycles
M1: C-C	765	F: AGATTTGTCCACGCCCACAT	56	1	40
		R: TCAAATTGGGAGATGCCAGCT			
M2: *LORF11*	584	F: GGGTTGCACAATCTTCTCAAA	55	1	45
		R: ACGTCCGTTTCTCCAGAATG			
M3: *meq*	1251	F: GAGGTTGGTGCTGGAATGTT	57	1.25	40
		R: AATGCCTTTAACCCTTTCCTTT			
M4: *ICP4* (prox.)	849	F: AAACCCCATTTTCGTGCAGC	56	1	40
		R: GCAAATGCGTTACCTGGAAA			
M5: *ICP4* (dist.)	482	F: GAGGAGGATGTCACCCTGAA	54	1	42
		R: CACAACCTCATCTCCACGAA			
M6: *pp38*	824	F: CAGAATCCACTCCCCCAACGACA	57	1	40
		R: CGAAGCAGAACACGAAGG			
M7: *UL36*	401	F: ACCGCCACTACCGTTACATC	55	1	40
		R: GCGCCTCGTCAAATATCC			
M8: *UL43*	340	F: TGGTACTCGGGCCAACTTTA	55	1	40
		R: CCGATGGTACCTTTGTTTTCA			

Two additional primers, internal to those provided in Table 3, were also employed for sequencing M3 (*meq*): F2, 5′-AGAAGACGCAGGAAGCAGAC-3′); R2, 5′-GGTACACGGCTCGGTAACAG-3′.

Viral density present in dust samples differed markedly according to sampling date, farm, and age of flock (Kennedy et al. 2017). Ideally, 10 μl of template would contain in excess of 10^4^ MDV genomes, although PCR amplicons for sequencing were still attainable from as few as 50–100 genomes.

### 2.4 Sanger sequencing

Initial Sanger sequencing was performed without cloning. This enabled greater numbers of samples to be examined rapidly and, through close scrutiny of the sequencing chromatograms, assessed for the presence of minor alleles (mixed populations). We then examined putative mixed populations more closely using cloning.

Amplification products were visualized on a 1.5 per cent agarose gel, the target amplicon excised and then purified using the EZNA Gel Extraction Kit (Omega Bio-tek, cat. no. D2500-02). Sanger sequencing was performed by the Pennsylvania State University Genomics Core Facility utilizing the same primers as used for DNA amplification (Table 3). However, given the *meq* gene’s amplicon size, the additional forward (5′-AGAAGACGCAGGAAGCAGAC-3′) and reverse (5′-GGTAC ACGGCTCGGTAACAG-3′) primers, internal to those described above, were also used for sequencing—thereby providing four overlapping sequences for consensus construction.

### 2.5 Cloning of the *meq* and *ICP4* (distal) markers for resolution of samples apparently containing multiple variants with different genotypes

When sequencing chromatograms indicated the presence of multiple variants at a marker region in a single sample, these samples were amplified according to the cycling conditions and primers detailed above using the GoTaq Green Master Mix (Promega, cat. no. M7122) which generates amplicons with the 3′A-tails necessary for cloning into the pGEM-T Easy Vector System II (Promega, cat. no. A1380). An overnight ligation (with vector to insert ratios of 1: 1) followed the conditions and concentrations recommended by the manufacturer, as did the subsequent transformation of pGEM-T vector (with insert) into JM109 competent cells. Transformants were plated onto LB/ampicillin/IPTG/X-Gal plates (each sample in duplicate) and allowed to incubate at 37°C overnight. White colonies of each sample were selected and screened (by standard PCR and gel visualization) to ensure the presence of the insert. Twelve colonies with inserts were subsequently grown overnight in LB at 37°C. Plasmids were extracted using the EZNA Plasmid DNA Mini Kit I (Omega Bio-tek, cat. no. D6943-02), their concentrations (150–300 ng/μl) determined using a Nanodrop 1000 (Thermo Scientific) and then submitted for Sanger sequencing in both directions, again utilizing the same primers listed above.

In our study area, all chickens on commercial farms are vaccinated against MDV, most commonly using the live attenuated bivalent vaccine, which is a combination of HVT and *Gallid herpesvirus 3* strain SB-1. None of our PCR primers amplify the DNA of those vaccine strains. A third vaccine strain, Rispens (CVI988) is used in layer birds and occasionally in broilers. One of the haplotypes defined by our eight markers is an exact match for the Rispens vaccine strain sequenced in GenBank. Moreover, this haplotype was only found on farms with a documented history of Rispens vaccination. We therefore refer to this haplotype as the ‘Rispens haplotype’, and we do not consider it to be wild-type virus. Note that some MDV haplotypes have the same allelic variants as Rispens at markers M2, M4, M5, M7, and M8 (see Fig. 1). The percentage of wild-type virus present in several dust samples that also apparently harbor the Rispens haplotype was determined by both qPCR and cloning. These methodologies provided similar ratios, with qPCR indicating 93, 68, and 7 per cent wild-type virus in three such samples and clone ratios indicating 86, 57, and 9 per cent, respectively.

### 2.6 Phylogenetic analysis of the deduced amino acid sequence of the *meq* gene

MDV isolates vary widely in pathotype (Witter 1997). Previous work has found phylogenetic separation between MDV pathotypes based on the *meq* gene (our marker M3) (Padhi and Parcells 2016). To explore whether different pathotypes of MDV might exist within our samples, we generated a phylogenetic tree using the Neighbor-Joining method (Saitou and Nei 1987). The analysis involved 95 complete Meq amino acid sequences (89 acquired from GenBank and 6 unique additions from this study: five variants found in Pennsylvania and a sixth from Arkansas). Evolutionary distances were computed using the Poisson correction method (Zuckerkandl and Pauling 1965) and are in units of amino acid substitutions per site. All positions containing gaps and missing data were eliminated. There was a total of 339 positions in the final dataset. Evolutionary analyses were conducted in MEGA7 (Kumar, Stecher, and Tamura 2016).

## 3. Results

Our initial genotyping approach used three marker regions (M2, M3, and M5, see Table 2) to assess the genetic diversity of 119 dust samples from 19 farms. These three markers revealed a total of eleven unique haplotypes. If we had used just a single marker, we would have detected at most six haplotypes in these samples, which demonstrates the benefits of multilocus typing. Out of the 119 samples analyzed, we found that 56 samples from 13 farms either had unique haplotypes or appeared to contain multiple variants at a single marker region, based on the visual examination of sequencing chromatograms. We therefore expanded our multilocus genotyping approach to include five additional markers (M1, M4, M6, M7, and M8). Table 4 shows the number of alleles—between two and six—that were identified by each individual marker region. In total, we were able to identify at least twelve haplotypes (Fig. 1). Three additional haplotypes may exist, because the two alleles of M4 cannot be definitively ‘linked’ to those three obtained for M5. The M5 marker was cloned for sixteen DNA samples and the M3 marker for a single DNA sample to resolve ambiguous sequences obtained from potentially mixed populations or, in a single instance, to look more closely at a haplotype that was only ever observed on a single farm.

**Table 4.** Number of SNPs present and MDV variants identified for each marker region.

Marker	Amplicon size	Number of SNPs previously identified among the ten fully sequenced MDV-1 strains	Number of SNPs identified in our samples	Number of variants identified in our samples
M1: C-C	765	11	1 (0 unique to our samples)	2: variant 1 = RB-1B, Md5, and 648A; variant 2 is unique.
M2: *LORF11*	584	7	2 (1 unique to our samples)	3: variant 1 = Rispens and strain 814 (both vaccine strains); variant 2 is unique; variant 3 = RB-1B, Md5, and 648A
M3: *meq*	1251	18	16 (7 unique to our samples)	5: all five variants unique to the full genome *meq* sequences. Variant 1 = 595, 549, L, RL, TK, and X (USA strains); variant 5 = 637 and 617A (USA strains); variants 2, 3, and 4 are unique among the 136+ *meq* sequences present in the databases
M4: *ICP4* (prox.)	849	8	4 (4 unique to our samples)	3: variant 1 is unique; variant 2 = Md5 and Rispens; variant 3 is unique
M5: *ICP4* (dist.)	482	9	5 (1 unique to our samples)	6: variants 1, 2, 4, and 5 are unique; variant 3 = RB-1B and Md5; variant 6 = Rispens, LMS, GA, and strain 814
M6: *pp38*	824	2	1 (0 unique to our samples)	2: variant 1 = LMS, GX0101, and CU-2; variant 2 = 648A, RB-1B, GA, Md5
M7: *UL36*	401	2	1 (0 unique to our samples)	2: variant 1 = 648A; variant 2 = other nine fully sequenced isolates
M8: *UL43*	340	6	1 (0 unique to our samples)	2: variant 1 = 648A; variant 2 = other nine fully sequenced isolates

None of the haplotypes described here exhibited 100 per cent identity to any of the fully sequenced strains present in GenBank (as of May 2018) with the exception of those from Pandey et al. (2016), which were collected from two of the same farms used in this study. The aligned sequences of each MDV variant and that of the Rispens haplotype are provided in the Supplementary Data.

**Figure 2.**
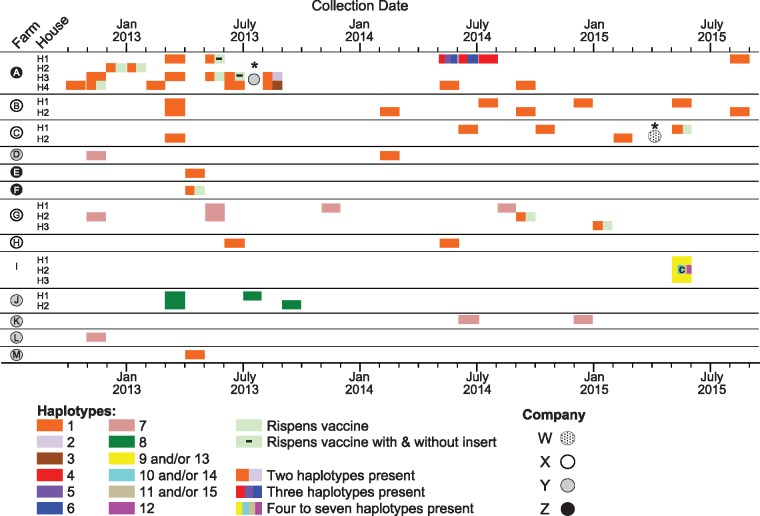
MDV haplotypes present on Pennsylvanian poultry farms between October 2012 and September 2015. Individual farms are identified as A–M and the presence of multiple poultry houses on these farms as H1, H2, etc. Individual colored bars represent different haplotypes as described in Fig. 1. Split color bars for an individual month indicate the presence of multiple haplotypes present at that time within a single dust sample. The pale green bar with a ‘–’ indicates the presence of two forms of the Rispens haplotype: one with its characteristic *meq* gene that contains an insert not typically present in wild-type virus and a second form that has lost this insert (see main text). ‘c’ denotes a sample that had M5 cloned. The company (W–Z) affiliated with each farm is indicated by dotted, white, gray, or black-filled circles, respectively. The asterisks indicate the points where farm A and farm C switched company affiliation.

Six of the original 19 farms provided just a single sampling point as MDV was only present once at amplifiable densities and indeed sequence data was only attainable for certain markers. According to the partial datasets obtained, these six farms harbored only haplotype 1, the haplotype which turned out to be the most prevalent across the thirteen remaining farms. The haplotypes present on those thirteen remaining ‘focal’ farms (A–M) sampled between October 2012 and September 2015 are shown in Fig. 2. All are commercial broiler farms, with the exceptions of farm I, which is a backyard flock and farm M, which raises broiler-breeder birds. At least twelve nonvaccine MDV haplotypes were detected. Two haplotypes were particularly prevalent: haplotype 1 was observed on nine farms and haplotype 7 on four farms. A haplotype identical to that of the Rispens vaccine (collated from sequences present in GenBank), which is occasionally used on broiler farms, was sometimes detected in mixed populations with wild-type virus, but it was never found alone (Fig. 2). Note that we cannot definitively say that observing this Rispens haplotype denotes the presence of the vaccine virus as opposed to wild-type virus, but we consider it likely (Fig. 1).

**Figure 3.**
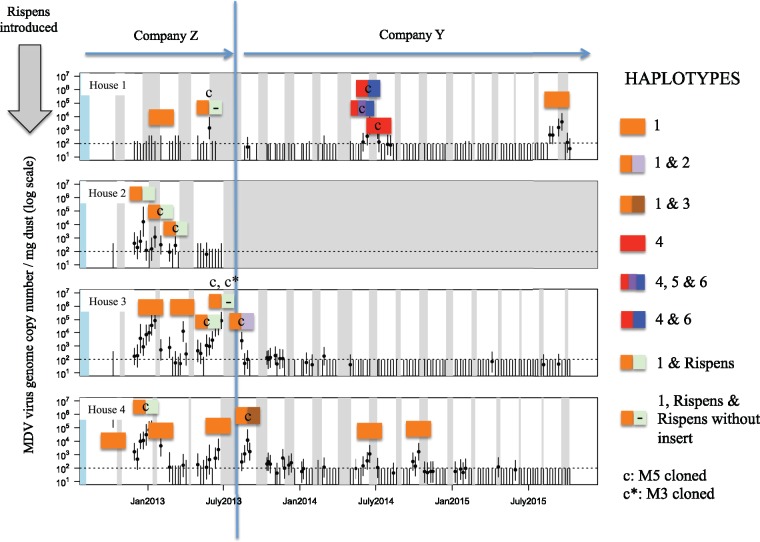
Prevalence, intensity, and diversity of MDV haplotypes present on farm A between October 2012 and September 2015. The *y*-axis indicates MDV virus genome copies/mg dust as determined by qPCR (see Kennedy et al. 2017). Data points show the log-mean virus concentration from sampled dust and the vertical error bars show 95 per cent confidence intervals of the mean (explained in detail in Kennedy et al. 2017). White intervals represent when a flock of birds was present and gray intervals when birds were absent. The dotted horizontal line marks the approximate limit of confident detection by qPCR (100 virions/genomes per mg dust). The vertical arrow labeled ‘Rispens introduced’ denotes that one flock prior to the commencement of sampling, birds on the farm had been Rispens vaccinated. The vertical solid line indicates the time point at which this farm changed company affiliation. Individual colored bars represent individual dust samples and the different haplotypes present therein as described in Fig. 1. Split color bars for an individual dust sample indicate the presence of multiple variants present at that time. The colored bars are centered above the dust sample haplotyped. The pale green bar with a ‘–’ indicates the presence of two forms of Rispens vaccine: one with its characteristic *meq* gene that contains an insert not typically present in wild-type virus and a second form with a *meq* gene that does not contain this insert (see main text). ‘c’ denotes a sample that had M5 cloned; ‘c*’ a sample that had M3 cloned.

On nine of the thirteen farms, only a single non-Rispens haplotype was found (Fig. 2): haplotype 1 on six farms; haplotype 7 on two; and haplotype 8 on one. On farms D and G, haplotype 7 was found during early collections and haplotype 1 was found during later sampling. On farm A (Fig. 3), we detected six wild-type haplotypes and the Rispens haplotype. On farm I, which was a backyard flock, we detected at least four haplotypes: at least three within a single dust sample and one more from a separate dust sample collected at the same time in the same building. In total, six samples contained more than one wild-type haplotype (four on farm A and two on backyard farm I). An additional ten samples contained haplotype 1 present with the Rispens haplotype.

**Figure 4.**
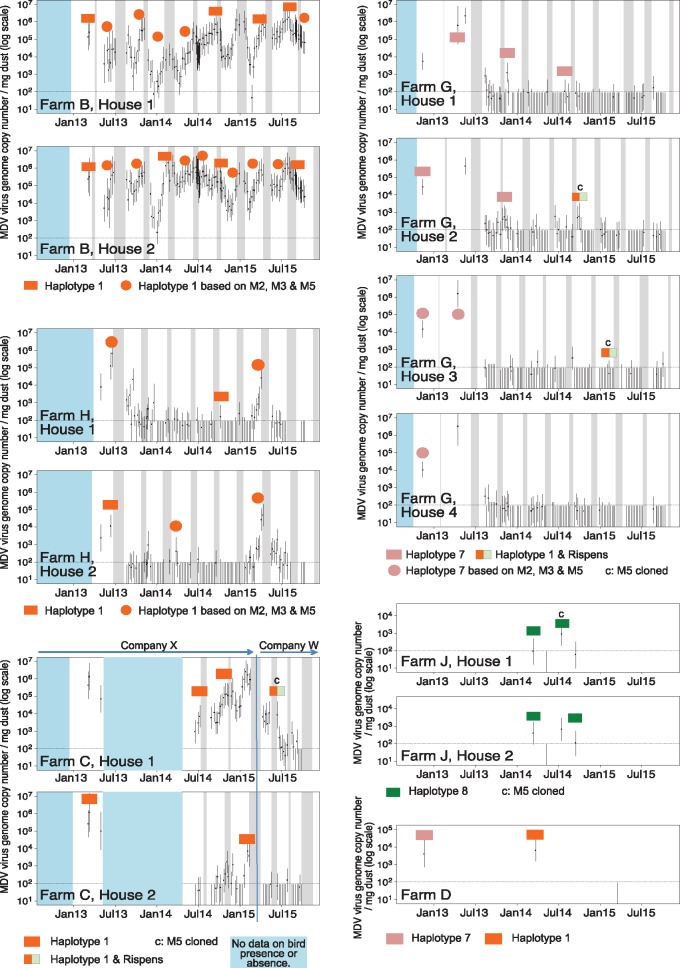
Prevalence, intensity, and diversity of MDV haplotypes present on farms B, C, D, G, H, and J between March 2013 and September 2015. Graph layouts and axes are as described in Fig. 3. Individual colored bars represent different haplotypes as described in Fig. 1. Additional data points (colored circles) with haplotype identification based on only three markers (M2, M3, and M5) are also shown. The blue blocks indicate time periods when there was no data on the presence or absence of birds.

The haplotypes detected on selected individual farms are shown in Figs 3 and 4, superimposed on the concentrations of wild type MDV present in dust reported by Kennedy et al. (2017). We know from personal communication with the grower that farm A used Rispens vaccination just prior to the commencement of sampling, and indeed a haplotype corresponding to the Rispens vaccine was observed in all four houses, but always in association with wild-type haplotype 1 (Fig. 3). Despite not using Rispens vaccination during the sample collection period, the Rispens haplotype continued to be detected until an extended break period between bird cohorts that coincided with the farm changing its company affiliation. Following that event, the Rispens haplotype was no longer detected, but five additional viral haplotypes appeared. A year later, none of those five haplotypes could be detected, and haplotype 1 reappeared. Two of the dust samples from farm A which were positive for both haplotype 1 and the Rispens haplotype contained an additional *meq* genotype: that of the Rispens vaccine, but without its characteristic 180 bp insertion. We believe this virus is a mutation in Rispens because the 3 bp deletion (CCA, see Supplementary Data) immediately upstream from the insertion site, and typically associated with its presence, was still observed.

On farms B, C, H, and J, a single haplotype apparently persisted over many months despite often dramatic fluctuations in viral densities in dust (Fig. 4, farms B, C, H, and J). On two other farms (D and G), haplotype 7 was initially detected but it was replaced by haplotype 1 or haplotype 1 and the Rispens haplotype (Fig. 4).

There was no obvious association between viral diversity and the breed of birds on a farm (Table 5). The most intensively sampled farm (farm A) had the highest diversity of viral strains (haplotypes 1–6) and that farm also utilized the greatest variety of birds. Prior to changing its company affiliation, Cobb, Cobb & Red Pedigree and Red Pedigree & Grey were raised on farm A. During this period, only haplotype 1 was observed (Fig. 3). After changing its company affiliation, only white birds were used (either Cobb, Hubbard × Ross, Cobb × Hubbard, or Hubbard) and five new unique-to-farm haplotypes appeared (haplotypes 2–6). The backyard farm (farm I) had Silkie birds in one house (containing viral haplotypes 9–12+) as well as unknown mixed-breed birds in two other houses (less diverse mixtures of the same viral haplotypes identified). At some point all of the other haplotypes described in the commercial farms were found on farms with Cobb birds (haplotypes 1–8). However, we cannot be sure that all of the viral haplotypes actually infected Cobb birds without taking samples directly from individual animals, because MDV can potentially persist in dust for months (Carrozza et al. 1973).

**Table 5.** Viral diversity and bird strains present on farms.

Farm	Bird strain								
	Red Pedigree & Grey	Cobb	Cobb & Red Pedigree	Cobb × Hubbard	Hubbard & Ross	Hubbard	Red Pedigree	Silkie	Unknown mixed breed
A	1	1–6	1	1	1, 4, 6	1			
B							1		
C				1			1		
D		1, 7							
E		1							
F	1								
G							1, 7		
H							1		
I								9–12+	9 and/or 13
J		8			8				
K							7		
L		7							
M					1				

Numbers represent viral haplotype; ‘&’ a mixed flock of two bird strains; ‘×’ a strain cross.

**Figure 5.**
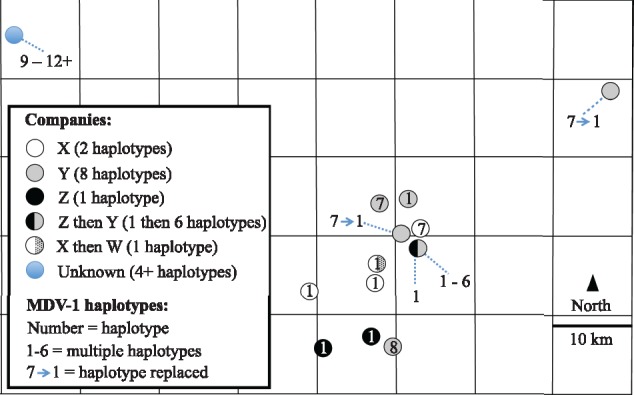
Spatial separation of the thirteen focal poultry farms surveyed in Pennsylvania indicating the company affiliation and MDV haplotypes identified. The farm in the top left is the backyard flock, where the birds were not associated with any company. The plotted area is approximately 50 × 80 km.

The spatial locations of the thirteen focal farms, the companies they are associated with, and the haplotypes recorded at each are shown in Fig. 5. The number of MDV variants was notably higher (eight haplotypes) on farms dealing with one of the four companies (company Y), although five of these haplotypes were identified only on farm A, the most intensively sampled farm (Fig. 5). The spatial location of individual farms revealed no marked patterns in terms of MDV diversity (Fig. 5) although haplotype 1 and haplotype 7 might be loosely separated by a SW/NE divide.

**Figure 6.**
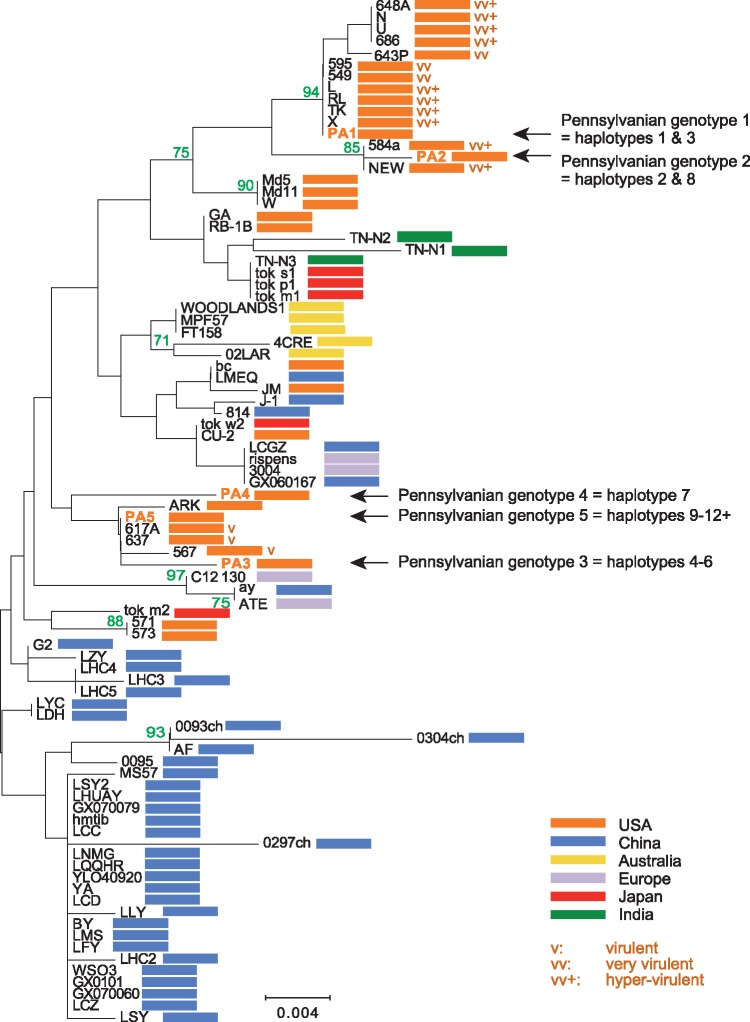
Phylogenetic analysis of MDV based on Meq amino acid sequences. The optimal tree with the sum of branch length = 0.209 is shown. The tree is drawn to scale, with branch lengths in the same units as those of the evolutionary distances used to infer the phylogenetic tree. The analysis involved 88 complete Meq amino acid sequences. Accession numbers of individual isolate sequences are provided in the Supplementary Data. All positions containing gaps and missing data were eliminated. There were a total of 339 positions in the final dataset. Colored bars indicate country of origin. PA1 to PA5 show the five Pennsylvanian genotypes and their haplotype associations identified in the current study. Where known, pathotypes of isolates present in clades occupied by our Pennsylvanian haplotypes are indicated by ‘v’ = virulent, ‘vv’ = very virulent, and ‘vv+’ = hyper-virulent.

In our samples, we found five distinct wild-type sequences of *meq* (marker M3, see Fig. 1 and Supplementary Data) among the 12+ haplotypes described. In Fig. 6, we show the phylogenetic relationship of these five Pennsylvanian *meq* alleles relative to those of 83 other sequenced isolates collected from around the world. Two notable patterns emerge. First, all of our Pennsylvania isolates fall into one of two clades, comprised only of other US isolates. Second, eight of our haplotypes (haplotypes 4–7 and 9–12) fall into the clade where every pathotyped virus is of type ‘v’ (3 isolates), and the other four haplotypes (haplotypes 1–3 and 8) fall into the clade where no pathotyped virus is of type ‘v’ (3 ‘vv’ and 9 ‘vv+’ isolates).

## 4. Discussion

### 4.1 General findings

We report a genetic analysis of archival MDV-positive samples collected on 19 Pennsylvanian farms over a period of 3 years. To our knowledge no study has previously attempted to describe the molecular epidemiology of MDV in the field using multiple genetic markers. We found that MDV diversity and strain dynamics can vary substantially within a relatively localized community, demonstrating the presence of haplotype variation on which selection may act.

These data demonstrate that a single MDV haplotype may be present on a poultry farm for several years, spanning many different flocks of birds, even if the density of MDV fluctuates substantially (e.g., Figs 2 and 4, farms B, C, H, and J). Indeed, virus presence in dust samples may fall to undetectable levels (as determined by qPCR assays), only for the same haplotype to reappear later (e.g., Fig. 4, farms C, G, H, and J).

At other farms, we found that multiple virus haplotypes can be present at the same time, and even within a single dust sample (Figs 2 and 3). Some farms alternatively had a single variant that appeared to be replaced by one or more alternatives (e.g., farms A, D, and G; Figs 3 and 4). In one case, the original haplotype eventually returned (farm A; Fig. 3). Whether this pattern was due to local extinction and reintroduction or persistence below PCR-detectable levels is presently unknown. However, the haplotype that reemerged was the most prevalent haplotype in our study, and previous theoretical work has indicated virus reintroduction rates might be quite high (Kennedy, Dunn, and Read 2018). We therefore find it highly plausible that the reemergence was due to a reintroduction event, but more conclusive evidence would likely require whole genome sequencing to determine whether these two isolates are indeed no more closely related to each other than to haplotype 1 isolates from nearby farms.

Twelve of our thirteen focal farms were commercial farms, with eleven rearing broiler chickens and one rearing broiler-breeder chickens. The dust sample that contained the most haplotypes (at least three) was one of five samples we obtained from the 13th farm, which contained a noncommercial backyard flock (haplotypes 9–12+). The birds present on this farm were kept in three houses, one with Silkie birds and the other two with mixed-breed birds. Notably, this farm was experiencing a confirmed outbreak of clinical Marek’s disease at the time of sample collection, whereas none of our other farms reported clinical disease while we were sampling them. It is unknown whether the large number of haplotypes is a cause or a consequence of the clinical disease outbreak, but further sampling and genotype analysis of clinical outbreaks is warranted to address this question in the future.

If we restrict our analysis to the eight haplotypes circulating on commercial farms, all eight haplotypes were found on the subset of six farms associated with company Y, while only two haplotypes were found on the four farms associated with company X, and only one haplotype was found on the three farms associated with company Z (note that this number excludes the haplotypes found on farm A only after it had changed affiliation to company Y) and similarly on the single farm associated with company W (Fig. 5). This pattern cannot be explained by differences in sampling depth—for example, many of our most intensively sampled farms exhibited very little diversity—and therefore suggests differences between companies in levels of pathogen diversity. Differences in MDV diversity between companies could be due to differences in biosecurity or animal husbandry protocols and practices.

Haplotype 1 was found consistently across the study period and was identified at some point on nine of the thirteen focal farms (Fig. 2). It was present on farms belonging to all three companies (Fig. 5) and on commercial farms using all seven breeds reared (Table 5). On six farms, it was the only haplotype ever found, including one farm where it apparently persisted over at least ten successive cohorts of birds (farm B, Fig. 4). We finished our study in September 2015, but we continued to sample occasionally on farm B for almost three more years. MDV was undetectable by qPCR between February 2017 and April 2018, and yet the same haplotype reappeared in May 2018 (data not shown). This suggests that haplotype 1 is a common MDV genotype in commercial chicken flocks in Pennsylvania. Notably, haplotype 1 clusters with highly virulent ‘vv’ and ‘vv+’ isolates based on *meq* (Fig. 6).

### 4.2 Limits on diversity detected

Our study is by no means an exhaustive analysis of MDV diversity. We limited our analysis to eight marker regions, which revealed a minimum of twelve haplotypes. Limitations in reconstructing haplotypes when cloning means that there may be as many as 15 wild-type haplotypes (plus that matching the Rispens vaccine) detected in our study (Fig. 1). However, even this larger number may be a gross underestimate for the total MDV diversity in central Pennsylvania. Haplotypes 2 and 3 were only ever identified in mixtures with haplotype 1 on farm A. Haplotype 2 differs from haplotype 1 at three markers (M3, M4, and M5) and haplotype 3 at two markers (M4 and M5). Given that haplotype 1 was previously present on the farm, a parsimonious conclusion was drawn by subtraction: that there was only a second haplotype present that exhibited all of these differences. However, it is also possible that these two mixtures might have been far more complex, with up to eight haplotypes present.

Our diversity survey is also limited by the total number of farms (19 in total, 13 focal) and the total number of dust samples processed (119). In addition, almost all of our sampling came from commercial poultry houses. Yet between three and six haplotypes were detected in a single dust sample from a backyard poultry farm (plus an additional haplotype from a second dust sample collected at the same time in the same house). Only eight haplotypes were observed on all of the commercial farms. The backyard poultry farm was not randomly selected for our study, but rather chosen because the birds on the farm were experiencing clinical Marek’s disease at the time the samples were collected. Nevertheless, these results are at least suggestive that backyard flocks may harbor more MDV diversity than commercial poultry flocks. More samples from backyard flocks, particularly those not currently suffering from clinical Marek’s disease, would be needed to further explore this hypothesis. Notably, all of the *meq* variants found on this noncommercial farm phylogenetically cluster with relatively less virulent ‘v’ isolates of MDV (Fig. 6).

In addition, we may have missed diversity present even within the samples that we did process. That is because we only performed cloning on samples with minor alleles that were obvious by Sanger sequencing, and these chromatograms are unable to reveal the presence of minor alleles below ≈15–20 per cent (Rohlin et al. 2009). Moreover, even when we did clone the marker regions from these dust samples, the sensitivity of such an approach is limited by the number of clones examined. In our case we sequenced twelve clones per sample, meaning that we could easily miss variants rarer than about 10 per cent. It would be interesting to examine some of our more diverse samples using deep sequencing, to explore whether other rare variants are present. This approach could reveal additional sites of diversity genome-wide. However, due to the large size (≈177 kb) of the viral genome relative to the length of most short-read (i.e., Illumina) sequencers, it would be difficult if not impossible to definitively link genotype variants at spatially separated loci.

### 4.3 Discriminatory power of the markers used

Among our panel of markers, M5, which encompasses a distal region of the *ICP4* gene, proved to have the greatest discriminatory ability for the strains present on these Pennsylvanian poultry farms, identifying six distinct forms. Coupling this marker with M3, which covers the *meq* gene, increased our resolution to detecting ten wild-type haplotypes with just two markers. The inclusion of M2 or M6 identifies one additional haplotype and inclusion of M1 adds another. Between 0 and 3 additional haplotypes could be gained by adding M4. M7 and M8 provided no greater resolution than M3 and M5 together. Of course, the respective importance of the individual markers might differ greatly with other populations.

### 4.4 Diversity previously determined using individual gene markers

Numerous authors have investigated individual MDV genes, in a search for those associated with traits, such as oncogenicity and virulence. Three genes that have received particular attention in this respect are *meq*, *pp38*, and *vIL-8*. The first two are our respective markers M3 and M6 (M6 is only a partial region of *pp38*). We did not sequence vIL-8 given prior reports that this gene is highly conserved in US samples (Tian et al. 2011).

We found two alleles of *pp38* (M6) in addition to the Rispens-like allele. Ten of twelve haplotypes contained the same genotype identified by Tian et al. (2011) in their Chinese field isolates (ggG coding for Glycine at amino acid 109, see Supplementary Data). Only a minority of our haplotypes (2 of 12) contained an alternative SNP (ggA coding for Glutamate), that was previously thought to be an indicator of MDV isolates collected from the USA (Tian et al. 2011). Our data demonstrate that this *pp38* SNP is not indicative of US-derived strains, at least in Pennsylvania (see Supplementary Data and Fig. 1).

We found five alleles of *meq* (M3) in addition to the Rispens-like allele. The *meq* gene has received particular attention in previous studies, because it is a major oncogene (Shamblin et al. 2004; Tian et al. 2011; Zhang et al. 2011; Renz et al. 2012), and it may also be a marker for virulence (Padhi and Parcells 2016). Previous work on *meq* gene diversity has generated contrasting conclusions that are associated with the geographic location of sampling. In China, Zhang et al. (2011) sequenced the *meq* gene of 19 MDV isolates collected from five provinces between 2006 and 2008. Of these, eight shared 100 per cent identity, including samples from locations several thousand miles apart. In Australia, Renz et al. (2012) similarly found little sequence variation among the *meq* genes of six MDV isolates, despite their wide temporal and geographical separation. This lack of *meq* gene diversity also appeared in Japanese (Murata et al. 2013), Iraqi (Wajid et al. 2013), and Egyptian (Hassanin, Abdallah, and El-Araby 2013) isolates. In contrast, studies in the USA and Poland have found that the *meq* gene has high genetic diversity and power to discriminate between MDV strains (USA: Shamblin et al. 2004; Poland: Wozniakowski and Samorek-Salamonowicz 2014). Consistent with prior studies in the USA, we found five alleles of the *meq* gene within little more than 50 miles.

The five *meq* alleles that we detected cluster into two phylogenetic clades. Three alleles fall in a clade where all pathotyped virus isolates are type ‘v’ and two alleles fall into a clade where all pathotyped virus isolates are type ‘vv’ or ‘vv+’. While we cannot pathotype isolates based on phylogeny alone, this pattern suggests that multiple pathotypes may simultaneously coexist in Pennsylvania, and even within a single farm (farm A, houses 1 and 4; see Figs 3 and 6). Whether differences in pathotype explain the haplotype replacements seen on farms D and G (Fig. 4) is an open question. Nevertheless, we think it worthwhile to note that haplotype 1, the most common haplotype on vaccinated commercial farms, clusters with highly virulent ‘vv’ and ‘vv+’ isolates, whereas the haplotypes that appeared on the unvaccinated backyard flock all cluster with the relatively less virulent ‘v’ isolates. Coupled with previous studies that have shown that Marek’s disease vaccines may enhance the fitness of hypervirulent MDV isolates (Atkins et al. 2013a,b; Read et al. 2015; Rozins and Day 2016), our data are consistent with the possibility that vaccine-driven selection is maintaining hypervirulent strains in the field.

### 4.5 Coda

This Sanger sequencing-based investigation of the epidemiology of MDV within the Pennsylvanian poultry industry provides the first multilocus data on localized spatial and temporal diversity in MDV. Given the conserved nature of the MDV genome and the limitations of discriminating between viral isolates based on a small panel of markers, our findings are likely to be a conservative estimate of the true diversity present. Nevertheless, employing multiple markers greatly improved the resolution of distinct viral genotypes over a single best marker. Substantial advances will be made when whole genome sequencing can be bought to bear on these samples. These data demonstrate the need to overcome financial and technical hurdles associated with whole-genome molecular epidemiology for MDV.

## Supplementary Material

vey042_Supplementary_Data
