# Supplementary material for: Molecular epidemiology of Marek’s disease virus in central Pennsylvania, USA
Source: Virus Evol. 2019 Apr 23;5(1):vey042. doi: 10.1093/ve/vey042 (PMC6478013; doi:10.1093/ve/vey042)
Supplement: vey042_Supplementary_Data [file vey042_supplementary_data.pdf]

Supplementary data.

Sequence alignments of Pennsylvanian MDV and Rispens vaccine genotypes for the eight markers employed in the study.

MARKER NO. 1: Partial UL49.5 and UL50 genes.

```
Rispens         AGATTTGTCCACGCCCACATAATTTACCACTCCTCTTTAAACATATCTGCGGTGAATAGT
PA genotype 1   AGATTTGTCCACGCCCACATAATTTACCACTCCTCTTTAAACATATCTGCGGTGAATAGT
PA genotype 2   AGATTTGTCCACGCCCACATAATTTACCACTCCTCTTTAAACATATCTGCGGTGAATAGT
                ************************************************************

Rispens         CGAAAGCATGCGTGATACGAACCCGCTAAGAGGGCGGAGATTACCGCCACCAGACCCAGA
PA genotype 1   CGAAAGCATGCGTGATACGAACCCGCTAAGAGGGCGGAGATTACCGCCACCAGACCCATA
PA genotype 2   CGAAAGCATGCGTGATACGAACCCGCTAAGAGGGCGGAGATTACCGCCACCAGACCCATA
                ********************************************************** *

Rispens         TAAAACAGCACAGAAAACCCAGATGAAAAAGCTATTGACACGCCTACTGCAGAACACGTA
PA genotype 1   TAAAACAGCACAGAAAACCCAGATGAAAAAGCTATTGACACGCCTACTGCAGAACACGTA
PA genotype 2   TAAAACAGCACAGAAAACCCAGATGAAAAAGCTATTGACACGCCTACTGCAGAACACGTA
                ************************************************************

Rispens         GACTCCCAAAAATCTCCCATACTCGTGATTGAACTTCCCCAATCGACAAACGTCGCTTGC
PA genotype 1   GACTCCCAAAAATCTCCCATACTTGTGATTGAACTTCCCCAATCGACAAACGTCGCTTGC
PA genotype 2   GACTCCCAAAAATCTCCCATACTTGTGATTGAACTTCCCCAATCGACAAACGTCGCTTGC
                *********************** ************************************

Rispens         GAAGTGGCAATCAAAATAGCAACGCCGATAACTAAACTACATACTGCATTATGGATGTCC
PA genotype 1   GAAGTGGCAATCAAAATAGCAACGCCGATAACTAAACTACAGACTGCATTATGAATGTCC
PA genotype 2   GAAGTGGCAATCAAAATAGCAACGCCGATAACTAAACTACAGACTGCATTATGAATGTCC
                ***************************************** *********** ******

Rispens         ATGAGTCCCATCCTCGTCGAGATCGTGATTTGGAGAATATAACGTTGGAAGCACTAACAG
PA genotype 1   ATGAGTCCCATCCTCGTCGAGATCGTGATTTGGAGAATATAACGTTGGAAGCACTAACAG
PA genotype 2   ATGAGTCCCATCCTCGTCGAGATCGTGATTTGGAGAATATAACGTTGGAAGCACTAACAG
                *C**********************************************G***********

Rispens         TTCCCTGGCGTCTGCAATTCCGTGTCGATGAAGCGTTATATGCAGTAAACCCAAACGGTT
PA genotype 1   TTCCCTGGCGTCTGCAATTCCGTGTCGATGAAGCGTTATATGCAGTAAACCCAAACGGTT
PA genotype 2   TTCCCTGGCGTCTGCAATTCCGTGTCGATGAAGCGTTATATGCAGTAAACCCAAACGGTT
                ***********T************************************************

Rispens         GGACCTGTTATATAGAAGAGCGTGATCAGCGTTGTCTTCGCGTTACCAACAATTGTGTGA
PA genotype 1   GGACCTGTTATATAGAAGAGCGTGATCAGCGTTGTCTTCGCGTTACCAACAATTGTGTGA
PA genotype 2   GGACCTGTTATATAGAAGAGCGTGATCAGCGTTGTCTTCGCGTTACCAACAATTGTGTGA
                ********************************************************A***

Rispens         TCTCGTTACTGAAATCGGATTTAAAGCGCAAATATCAAACGTGTACATTGAATATCGGAA
PA genotype 1   TCTCGTTACTGAAATCGGATTTAAAGCGCAGATATCAAACGTGTACATTGAATATCGGAA
PA genotype 2   TCTCGTTACTGAAATCGGATTTAAAGCGCAAATATCAAACGTGTACATTGAATATCGGAA
                ****************************** *****************************

Rispens         TACGAGTTGCCGTTCCTCAGAATTACGTGGTCATTTTGGCAAAGTTAACGGATCCAGACC
PA genotype 1   TACGAGTTGCCGTTCCTCAGAATTACGTGGTCATTTTGGCAAAGTTAACGGATCCAGACC
PA genotype 2   TACGAGTTGCCGTTCCTCAGAATTACGTGGTCATTTTGGCAAAGTTAACGGATCCAGACC
                *********************************************C**************
```

```
Rispens         CAACTTCTCGAGGTATACCCATCATTCAAGTCGCCAATGGATTGATAGATTCAGGATATA
PA genotype 1   CAACTTCTCGAGGTATACCCATCATTCAAGTCGCCAATGGATTGATAGATTCAGGATATA
PA genotype 2   CAACTTCTCGAGGTATACCCATCATTCAAGTCGCCAATGGATTGATAGATTCAGGATATA
                ********************T***************************************

Rispens         GGGGATCTATCCGAGCAGTATTGTTTTTTGAAAAATCATGTATCATTCCAAAAAATGGGC
PA genotype 1   GGGGATCTATCCGAGCAGTATTGTTTTTTGAAAAATCATGTATCATTCCAAAAAATGGGC
PA genotype 2   GGGGATCTATCCGAGCAGTATTGTTTTTTGAAAAATCATGTATCATTCCAAAAAATGGGC
                ************************************************************

Rispens         TGGCTATTCGTTTATCACTAGTAAAGCTGGCATCTCCCAATTTGA
PA genotype 1   TGGCTATTCGTTTATCACTAGTAAAGCTGGCATCTCCCAATTTGA
PA genotype 2   TGGCTATTCGTTTATCACTAGTAAAGCTGGCATCTCCCAATTTGA
                *********************************************
```

Nucleotide in blue indicates the single SNP discriminating PA genotypes 1 and 2. Red nucleotides indicate additional previously recorded SNP sites among the ten strains with fully sequenced genomes. Gaps among asterisks indicate SNP differences between PA MDV-1 and Rispens vaccine strain genotypes. Accession numbers: PA1, MK084981; PA2, MK084982.

MARKER NO. 2: partial LORF11 gene.

```
PA genotype 1   GGGTTGCACAATCTTCTCAAAAAAGGTTTGATCAATGCAACTGCACCGCATCTCGACACA
PA genotype 2   GGGTTGCACAATCTTCTCAAAAAAGGTTTGATCAATGCAACTGCACCGCATCTCGACACA
PA genotype 3   GGGTTGCACAATCTTCTCAAAAAAGGTTTGATCAATGCAACTGCACCGCATCTCGACACA
                ************************************************************

PA genotype 1   TGTAACGGGGGCATAGTTGAACGTGCAACAGGTCTTTCTGTAAACAAATCTAGACGTATA
PA genotype 2   TGTAACGGGGGCATAGTTGAACGTGCAACAGGTCTTTCTGTAAACAAATCTAGACGTATA
PA genotype 3   TGTAACGGGGGCATAGTTGAACGTGCAACAGGTCTTTCTGTAAACAAATCTAGACGTATA
                *******************************************G***********A****

PA genotype 1   CGCCGGGAGCTGCGATACTGTATATTATGTTCGGCTGTATACGTAGAATGACTACGATGG
PA genotype 2   CGCCGGGAACTGCGATACTGTATATTATGTTCGGCTGTATACGTAGAATGACTACGATGG
PA genotype 3   CGCCGGGAGCTGCGATACTGTATATTATGTTCGGCTGTATACGTAGAATGACTACGATGG
                ******** ***************************************************

PA genotype 1   AGCCAATCATCCCATGTGCCAGTAAAGTACATTATAGGTGGAACTTTTTTTCTTTGCCTC
PA genotype 2   AGCCAATCATCCCATGTGCCAGTGAAGTACATTATAGGTGGAACTTTTTTTCTTTGCCTC
PA genotype 3   AGCCAATCATCCCATGTGCCAGTGAAGTACATTATAGGTGGAACTTTTTTTCTTTGCCTC
                *********************** ************************************

PA genotype 1   ACCTTAGAAGTGATGGTGGTACTATCCCCACTGATGTGTAAATTGAGGGTTTCTTTCCAG
PA genotype 2   ACCTTAGAAGTGATGGTGGTACTATCCCCACTGATGTGTAAATTGAGGGTTTCTTTCCAG
PA genotype 3   ACCTTAGAAGTGATGGTGGTACTATCCCCACTGATGTGTAAATTGAGGGTTTCTTTCCAG
                ************************************************************

PA genotype 1   GGTTTTAGTTTTTCGGATATTAACATATCGTTTGCCCGTCGACAACACTCTTCAACTACC
PA genotype 2   GGTTTTAGTTTTTCGGATATTAACATATCGTTTGCCCGTCGACAACACTCTTCAACTACC
PA genotype 3   GGTTTTAGTTTTTCGGATATTAACATATCGTTTGCCCGTCGACAACACTCTTCAACTACC
                *********************************************T***********C**

PA genotype 1   CATTTTAAATCGTCTAAGTACACTTCAGATAATTCCTTGACATAGCTTTCATCTGCATCG
PA genotype 2   CATTTTAAATCGTCTAAGTACACTTCAGATAATTCCTTGACATAGCTTTCATCTGCATCG
```

```
PA genotype 3   CATTTTAAATCGTCTAAGTACACTTCAGATAATTCCTTGACATAGCTTTCATCTGCATCG
                ***********************************************************A

PA genotype 1   CAATCACAACCATTCGGAAAATAATTGCCCTGATTATTATGTGTAGCTGCGGAACCATCA
PA genotype 2   CAATCACAACCATTCGGAAAATAATTGCCCTGATTATTATGTGTAGCTGCGGAACCATCA
PA genotype 3   CAATCACAACCATTCGGAAAATAATTGCCCTGATTATTATGTGTAGCTGCGGAACCATCA
                ***************************T********************************

PA genotype 1   AGCTCCATTTTATGATATAAGCGAGATACAGTATCCCTGGTATAACGATAACAGGGGGCA
PA genotype 2   AGCTCCATTTTATGATATAAGCGAGATACAGTATCCCTGGTATAACGATAACAGGGGGCA
PA genotype 3   AGCTCCATTTTATGATATAAGCGAGATACAGTATCCCTGGTATAACGATAACAGGGGGCA
                ************************************************************

PA genotype 1   ATATCCTTACTAGGGTATAACATGCATTCTGGAGAAACGGACGT
PA genotype 2   ATATCCTTACTAGGGTATAACATGCATTCTGGAGAAACGGACGT
PA genotype 3   ATATCCTTACTAGGGTATAACATGCATTCTGGAGAAACGGACGT
                ********************************************
```

Nucleotides in blue indicate the two SNP sites discriminating the three PA genotypes. Red nucleotides indicate additional previously recorded SNP sites among the ten strains with fully sequenced genomes. Rispens vaccine sequence is identical to PA genotype no. 1. Accession numbers: PA1, MK084983; PA2, MK084984; PA3, MK084905.

MARKER NO. 3: Full meq gene.

```
Rispens       ATGTCTCAGGAGCCAGAGCCGGGCGCTATGCCCTACAGTCCCGCTGACGATCCGTCCCCC
Rispens-      ATGTCTCAGGAGCCAGAGCCGGGCGCTATGCCCTACAGTCCCGCTGACGATCCGTCCCCC
PA genotype 2 ATGTCTCAGGAGCCAGAGCCGGGCGCTATGCCCTACAGTCCCGCTGACGATCCGTCCCCC
PA genotype 1 ATGTCTCAGGAGCCAGAGCCGGGCGCTATGCCCTACAGTCCCGCTGACGATCCGTCCCCC
PA genotype 4 ATGTCTCAGGAGCCAGAGCCGGGCGCTATGCCCTACAGTCCCGCTGACGATCCGTCCCCC
PA genotype 5 ATGTCTCAGGAGCCAGAGCCGGGCGCTATGCCCTACAGTCCCGCTGACGATCCGTCCCCC
PA genotype 3 ATGTCTCAGGAGCCAGAGCCGGGCGCTATGCCCTACAGTCCCGCTGACGATCCGTCCCCC
              ************************************************************

Rispens       CTCGATCTTTCTCTCGGGTCGACTTCGAGACGGAAAAAAAGGAAAAGTCACGACATCCCC
Rispens-      CTCGATCTTTCTCTCGGGTCGACTTCGAGACGGAAAAAAAGGAAAAGTCACGACATCCCC
PA genotype 2 CTCGATCTTTCTCTCGGGTCGACTTCGAGACGGAAAAAAAGGAAAAGTCACGACATCCCC
PA genotype 1 CTCGATCTTTCTCTCGGGTCGACTTCGAGACGGAAAAAAAGGAAAAGTCACGACATCCCC
PA genotype 4 CTCGATCTTTCTCTCGGGTCGACTTCGAGACGGAAAAAAAGGAAAAGTCACGACATCCCC
PA genotype 5 CTCGATCTTTCTCTCGGGTCGACTTCGAGACGGAAAAAAAGGAAAAGTCACGACATCCCC
PA genotype 3 CTCGATCTTTCTCTCGGGTCGACTTCGAGACGGAAAAAAAGGAAAAGTCACGACATCCCC
              ************************************************************

Rispens       AACAGCCCCTCCAAACACCCCTTCCCTGACGGCCTATCTGAGGAGGAGAAACAGAAGCTG
Rispens-      AACAGCCCCTCCAAACACCCCTTCCCTGACGGCCTATCTGAGGAGGAGAAACAGAAGCTG
PA genotype 2 AACAGCCCCTCCAAACACCCCTTCCCTGACGGCCTATCTGAGGAGGAGAAACAGAAGCTG
PA genotype 1 AACAGCCCCTCCAAACACCCCTTCCCCGACGGCCTATCTGAGGAGGAGAAACAGAAGCTG
PA genotype 4 AACAGCCCCTCCAAACACCCCTTCCCTGACGGCCTATCTGAGGAGGAGAAACAGAAGCTG
PA genotype 5 AACAGCCCCTCCAAACACCCCTTCCCCGACGGCCTATCTGAGGAGGAGAAACAGAAGCTG
PA genotype 3 AACAGCCCCTCCAAACACCCCTTCCCCGACGGCCTATCTGAGGAGGAGAAACAGAAGCTG
              ************************** *********************************

Rispens       GAAAGGAGGAGAAAAAGGAATCGTGACGCCTCTCGGAGAAGACGCAGGGAGCAGACGGAC
Rispens-      GAAAGGAGGAGAAAAAGGAATCGTGACGCCTCTCGGAGAAGACGCAGGGAGCAGACGGAC
PA genotype 2 GAAAGGAGGAGAAAAAGGAATCGTGACGCCGCTCGGAGAAGACGCAGGAAGCAGACGGAC
PA genotype 1 GAAAGGAGGAGAAAAAGGAATCGTGACGCCGCTCGGAGAAGACGCAGGAAGCAGACGGAC
PA genotype 4 GAAAGGAGGAGAAAAAGGAATCGTGACGCCGCTCGGAGAAGACGCAGGGAGCAGACGTAC
```

```
PA genotype 5 GAAAGGAGGAGAAAAAGGAATCGTGACGCCGCTCGGAGAAGACGCAGGGAGCAGACGTAC
PA genotype 3 GAAAGGAGGAGAAAAAGGAATCGTGACGCCGCTCGGAGAAGACGCAGGGAGCAGACGTAC
              ****************************** ***************** ******** **

Rispens       TATGTAGACAAACTCCATGAAGCATGTGAAGAGCTGCAGAGGGCCAATGAACACCTACGT
Rispens-      TATGTAGACAAACTCCATGAAGCATGTGAAGAGCTGCAGAGGGCCAATGAACACCTACGT
PA genotype 2 TATGTAGACAAACTCCATGAAGCATGTGAAGAGCTGCAGAGGGCCAATGAACACCTACGT
PA genotype 1 TATGTAGACAAACTCCATGAAGCATGTGAAGAGCTGCAGAGGGCCAATGAACACCTACGT
PA genotype 4 TATGTAGACAAACTCCATGAAGCATGTGAAGAGCTGCAGAGGGCCAATGAACACCTACGT
PA genotype 5 TATGTAGACAAACTCCATGAAGCATGTGAAGAGCTGCAGAGGGCCAATGAACACCTACGT
PA genotype 3 TATGTAGACAAACTCCATGAAGCATGTGAAGAGCTGCAGAGGGCCAATGAACACCTACGT
              ************************************************************

Rispens       AAGGAAATTCGAGATCTAAGGACTGAGTGCACGTCCCTGCGTGTACAGTTGGCTTGTCAT
Rispens-      AAGGAAATTCGAGATCTAAGGACTGAGTGCACGTCCCTGCGTGTACAGTTGGCTTGTCAT
PA genotype 2 AAGGAAATTCGAGATCTAAGGACTGAGTGCACGTCCCTGCGTGTACAGTTGGCTCGTCAT
PA genotype 1 AAGGAAATTCGAGATCTAAGGACTGAGTGCACGTCCCTGCGTGTACAGTTGGCTCGTCAT
PA genotype 4 AAGGAAATTCGAGATCTAAGGACTGAGTCCACGTCCCTGCGTGTACAGTTGGCTTGTCAT
PA genotype 5 AAGGAAATTCGAGATCTAAGGACTGAGTGCACGTCCCTGCGTGTACAGTTGGCTCGTCAT
PA genotype 3 AAGGAAATTCGAGATCTAAGGACTGAGTGCACGTCCCTGCGTGTACAGTTGGCTCGTCAT
              **************************** **************C********** *****

Rispens       GAGCCAGTTTGCCCTATGGCGGTACCCCTAACGGTGACCCTTGGACTGCTTACCACCCCG
Rispens-      GAGCCAGTTTGCCCTATGGCGGTACCCCTAACGGTGACCCTTGGACTGCTTACCACCCCG
PA genotype 2 GAGCCAGTTTGCCCTATGGCGGTACCCCTAACGGTGACCCTTGGACTGCTTACCACCCCG
PA genotype 1 GAGCCAGTTTGCCCTATGGCGGTACCCCTAACGGTGACCCTTGGACTGCTTACCACCCCG
PA genotype 4 GAGCCAGTTTGCCCTATGGCGGTACCCCTAACGGTGACCCTTGGACTGATTACCACCCCG
PA genotype 5 GAGCCAGTTTGCCCTATGGCGGTACCCCTAACGGTGACCCTTGGACTGCTTACCACCCCG
PA genotype 3 GAGCCAGTTTGCCCTATGGCGGTACCCCTAACGGTGACCCTTGGACTGCTTACCACCCCG
              ************************************************ *****G*****

Rispens       CACGATCCCGTTCCTGAACCTCCCATTTGCACTCCTCCACCTCCCTCACCGGATGAACCT
Rispens-      CACGATCCCGTTCCTGAACCTCCCATTTGCACTCCTCCACCTCCCTCACCGGATGAACCT
PA genotype 2 CACGATCCCGTTCCTGAACCTCCCATTTGCACTCCTCAACCTCCCTCACCGGATGAACCT
PA genotype 1 CACGATCCCGTTCCTGAACCTCCCATTTGCACTCCTCAACCTCCCTCACCGGATGAACCT
PA genotype 4 CACGATCCCGTTCCTGAACCTCCCATTTGCACTCCTCCACCTCCCTCACCGGATGAACCT
PA genotype 5 CACGATCCCGTTCCTGAACCTCCCATTTGCACTCCTCCACCTCCCTCACCGGATGAACCT
PA genotype 3 CACGATCCCGTTCCTGAACCTCCCATTTGCACTCCTCCACCTCCCTCACCGGATGAACCT
              ***A********************************* **********************

Rispens       AACGCTCCACATTGCTCCGGTTCCCAACCTCCTATCTGTACCCCCCCTCCTCCCGATACG
Rispens-      AACGCTCCACATTGCTCCGGTTCCCAACCTCCTATCTGTACCCCCCCTCCTCCCGATACG
PA genotype 2 AACGCTCCACATTGCTCCGGTTCCCAACCTCCTATCTGTACCCCCGCTCCTCCCGATACG
PA genotype 1 AACGCTCCACATTGCTCCGGTTCCCAACCTCCTATCTGTACCCCCGCTCCTCCCGATGCG
PA genotype 4 AACGCTCCACATTGCTCCGGTTCCCAACCTCCTATCTGTACCCCCCCTCCTCCCGATACG
PA genotype 5 AACGCTCCACATTGCTCCGGTTCCCAACCTCCTATCTGTACCCCCCCTCCTCCCGATACG
PA genotype 3 AACGCTCCACATTGCTCCGGTTCCCAACCTCCTATCTGTACCCCCCCTCCTCCCGATACG
              ********************************************* G********** **

Rispens       GAGGAACTTTGCGCCCAGCTCTGCTCGACCCCA---CCTCCCATCTCTACTCCCCATATT
Rispens-      GAGGAACTTTGCGCCCAGCTCTGCTCGACCCCA---------------------------
PA genotype 2 GAGGAGCTTTGCGCCCAGCTCTGCTCGACCCCACCA------------------------
PA genotype 1 GAGGAGCTTTGCGCCCAGCTCTGCTCGACCCCACCA------------------------
PA genotype 4 GAGGAACTTTGCGCCCAGCTCTGCTCGACCCCACCA------------------------
PA genotype 5 GAGGAACTTTGCGCCCAGCTCTGCTCGACCCCACCA------------------------
PA genotype 3 GAGGAACTTTGCGCCCAGCTCTGCTCGACCCCATCA------------------------
              ***** ***************************

Rispens       ATCTACGCTCCGGGGCCTTCCCCCCTCCAACCTCCTATCTGTACCCCCCCTCCTCCCGAT
Rispens-      ------------------------------------------------------------
PA genotype 2 ------------------------------------------------------------
PA genotype 1 ------------------------------------------------------------
PA genotype 4 ------------------------------------------------------------
PA genotype 5 ------------------------------------------------------------
PA genotype 3 ------------------------------------------------------------
```

```
Rispens       GCGGAGGAGCTTTGCGCCCAGCTCTGCTCGACCCCACCACCTCCCATCTCTACTCCCCAT
Rispens-      ------------------------------------------------------------
PA genotype 2 ------------------------------------------------------------
PA genotype 1 ------------------------------------------------------------
PA genotype 4 ------------------------------------------------------------
PA genotype 5 ------------------------------------------------------------
PA genotype 3 ------------------------------------------------------------

Rispens       ATTTTCTACGCTCCGGGGCTCTGCTCGACCCCACCACCTCCCATCTCTACTCCCCATATT
Rispens-      ------------------------------------CCTCCCATCTCTACTCCCCATATT
PA genotype 2 ------------------------------------CCTCCCATCTCTACTCCCCATATT
PA genotype 1 ------------------------------------CCTCCCATCTCTACTCCCCATATT
PA genotype 4 ------------------------------------CCTCCCATCTCTACTCCCCATATT
PA genotype 5 ------------------------------------CCTCCCATCTCTACTCCCCATATT
PA genotype 3 ------------------------------------CCTCCCATCTCTACTCCCCATATT
                                                  ************************

Rispens       ATCTACGCTCCGGGGCCTTCCCCCCTCCAACCTCCTATCTGTACCCCCCCTCCTCCCGAT
Rispens-      ATCTACGCTCCGGGGCCTTCCCCCCTCCAACCTCCTATCTGTACCCCCCCTCCTCCCGAT
PA genotype 2 ATCTACGCTCCGGGGCCTTCCCCCCTCCAACCTCCTATCTGTACCCCCGCTCCTCCCGAT
PA genotype 1 ATCTACGCTCCGGGGCCTTCCCCCCTCCAACCTCCTATCTGTACCCCCGCTCCTCCCGAT
PA genotype 4 ATCTACGCTCCGGGGCCTTCCCCCCTCCAACCTCCTATCTGTACCCCCGCTCCTCCCGAT
PA genotype 5 ATCTACGCTCCGGGGCCTTCCCCCCTCCAACCTCCTATCTGTACCCCCGCTCCTCCCGAT
PA genotype 3 ATCTACGCTCCGGGGCCTTCCCCCCTCCAACCTCCTATCTGTACCCCCGCTCCTCCCGAT
              ************************************************ ***********

Rispens       GCGGAGGAGCTTTGCGCCCAGCTCTGCTCGACCCCACCACCTCCCATCTGTACTCCCCAT
Rispens-      GCGGAGGAGCTTTGCGCCCAGCTCTGCTCGACCCCACCACCTCCCATCTGTACTCCCCAT
PA genotype 2 GCGGAGGAGCTTTGCGCCCAGCTCTGCTCGACCCCACCACCTCCCATCTGTACTCCCCAT
PA genotype 1 GCGGAGGAGCTTTGCGCCCAGCTCTGCTCGACCCCACCACCTCCCATCTGTACTCCCCAT
PA genotype 4 GCGGAGGAGCTTTGCGCCCAGCTCTGCTCGACCCCACCACCTCCCATCTGTACTCCCCAT
PA genotype 5 GCGGAGGAGCTTTGCGCCCAGCTCTGCTCGACCCCACCACCTCCCATCTGTACTCCCCAT
PA genotype 3 GTGGAGGAGCTTTGCGCCCAGCTCTGCTCGACCCCACCACCTCCCATCTGTACTCCCCAT
              * **********************************************************

Rispens       TCCCTCTTCTGCCCTCCCCAGCCTCCATCTCCGGAGGGCATCTTCCCTGCATTGTGTCCT
Rispens-      TCCCTCTTCTGCCCTCCCCAGCCTCCATCTCCGGAGGGCATCTTCCCTGCATTGTGTCCT
PA genotype 2 TCCCTCTTCTGCCCTCCCCAGCCTCCATCTCCGGAGGGCATCTTCCCTGCATTGTGTCCT
PA genotype 1 TCCCTCTTCTGCCCTCCCCAGCCTCCATCTCCGGAGGGCATCTTCCCTGCATTGTGTCCT
PA genotype 4 TCCCTCTTCTGCCCTCCCCAGCCTCCATCTCCGGAGGGCATCTTCCCTGCATTGTGTCCT
PA genotype 5 TCCCTCTTCTGCCCTCCCCAGCCTCCATCTCCGGAGGGCATCTTCCCTGCATTGTGTCCT
PA genotype 3 TCCCTCTTCTGCCCTCCCCAGCCTCCATCTCCGGAGGGCATCTTCCCTGCATTGTGTCCT
              **************************************A*********************

Rispens       GTTACCGAGCCGTGTACCCCTCCATCGCCGGGGACGGTTTACGCTCAGCTTTGTCCTGTT
Rispens-      GTTACCGAGCCGTGTACCCCTCCATCGCCGGGGACGGTTTACGCTCAGCTTTGTCCTGTT
PA genotype 2 GTTACCGAGCCGTGTACCCCTCCATCGCCGGGGACGGTTTACGCTCAGCTTTGTCCTGTT
PA genotype 1 GTTACCGAGCCGTGTACCCCTCCATCGCCGGGGACGGTTTACGCTCAGCTTTGTCCTGTT
PA genotype 4 GTTACCGAGCCGTGTGCCCCTCCATCGCCGGGGACGGTTTACGCTCAGCTTTGTCCTGTT
PA genotype 5 GTTACCGAGCCGTGTACCCCTCCATCGCCGGGGACGGTTTACGCTCAGCTTTGTCCTGTT
PA genotype 3 GTTACCGAGCCGTGTACCCCTCCATCGCCGGGGACGGTTTACGCTCAGCTTTGTCCTGTT
              *************** *********************************C**********

Rispens       GGCCAGGCTCCCCTTTTTACCCCATCTCCCCCACATCCGGCTCCGGAGCCGGAGAGGCTT
Rispens-      GGCCAGGCTCCCCTTTTTACCCCATCTCCCCCACATCCGGCTCCGGAGCCGGAGAGGCTT
PA genotype 2 GGCCAGGTTCCCCTTTTTACCCCATCTCCCCCACATCCGGCTCCGGAGCCGGAGAGGTTT
PA genotype 1 GGCCAGGCTCCCCTTTTTACCCCATCTCCCCCACATCCGGCTCCGGAGCCGGAGAGGCTT
PA genotype 4 GGCCAGGCTCCCCTTTTTACCCCATCTCCCCCACATCCGGCTCCGGAGCCGGAGAGGCTT
PA genotype 5 GGCCAGGCTCCCCTTTTTACCCCATCTCCCCCACATCCGGCTCCGGAGCCGGAGAGGCTT
PA genotype 3 GGCCAGGCTCCCCTTTTTACCCCATCTCCCCCACATCCGGCTCCGGAGCCGGAGAGGCTT
              ******* ************************************************* **
```

```
Rispens        TATGCTCGTCTTACCGAGGATCCCGAACAGGATTCCTTGTATTCGGGCCAGATTTATATT
Rispens-       TATGCTCGTCTTACCGAGGATCCCGAACAGGATTCCTTGTATTCGGGCCAGATTTATATT
PA genotype 2  TATGCTCGTCTTACCGAGGATCCCGAACAGGATTCCTTGTATTCGGGCCAGATTTATACT
PA genotype 1  TATGCTCGTCTTACCGAGGATCCCGAACAGGATTCCTTGTATTCGGGCCAGATTTATATT
PA genotype 4  TATGCTCGTCTTACCGAGGATCCCGAACAGGATTCCTTGTATTCGGGCCAGATTTATATT
PA genotype 5  TATGCTCGTCTTACCGAGGATCCCGAACAGGATTCCTTGTATTCGGGCCAGATTTATATT
PA genotype 3  TATGCTCGTCTTACCGAGGATCCCGAACAGGATTCCTTGTATTCGGGCCAGATTTATATT
               ********************************************************** *

Rispens        CAGTTTCCCTCGGATATTCAGTCTACGGTCTGGTGGTTTCCAGGTGACGGGAGACCCTGA
Rispens-       CAGTTTCCCTCGGATATTCAGTCTACGGTCTGGTGGTTTCCAGGTGACGGGAGACCCTGA
PA genotype 2  CAGTTTCCCTCGGATACTCAGTCTACGGTCTGGTGGTTTCCAGGTGACGGGAGACCCTGA
PA genotype 1  CAGTTTCCCTCGGATACTCAGTCTACGGTCTGGTGGTTTCCAGGTGACGGGAGACCCTGA
PA genotype 4  CAGTTTCCCTCGGATACTCAGTCTACGGTCTGGTGGTTTCCAGGTGACGGGAGACCCTGA
PA genotype 5  CAGTTTCCCTCGGATACTCAGTCTACGGTCTGGTGGTTTCCAGGTGACGGGAGACCCTGA
PA genotype 3  CAGTTTCCCTCGGATACTCAGTCTACGGTCTGGTGGTTTCCAGGTGACGGGAGACCCTGA
               **************** *******************************************
```

Nucleotides in blue indicate the sixteen SNP sites discriminating the five PA variants. Red nucleotides indicate additional previously recorded SNP sites among the ten strains with fully sequenced genomes. Gaps among asterisks indicate SNP differences between PA variants and Rispens vaccine strain. Accession numbers: PA1, MK040409; PA2, MK040410; PA3, MK040411; PA4, MK040412; PA5, MK040413.

MARKER NO. 3: meq gene deduced amino acid sequence.

```
PA genotype 2   MSQEPEPGAMPYSPADDPSPLDLSLGSTSRRKKRKSHDIPNSPSKHPFPDGLSEEEKQKL
PA genotype 4   MSQEPEPGAMPYSPADDPSPLDLSLGSTSRRKKRKSHDIPNSPSKHPFPDGLSEEEKQKL
PA genotype 1   MSQEPEPGAMPYSPADDPSPLDLSLGSTSRRKKRKSHDIPNSPSKHPFPDGLSEEEKQKL
PA genotype 3   MSQEPEPGAMPYSPADDPSPLDLSLGSTSRRKKRKSHDIPNSPSKHPFPDGLSEEEKQKL
PA genotype 5   MSQEPEPGAMPYSPADDPSPLDLSLGSTSRRKKRKSHDIPNSPSKHPFPDGLSEEEKQKL
Rispens         MSQEPEPGAMPYSPADDPSPLDLSLGSTSRRKKRKSHDIPNSPSKHPFPDGLSEEEKQKL
                ************************************************************

PA genotype 2   ERRRKRNRDAARRRRRKQTDYVDKLHEACEELQRANEHLRKEIRDLITECTSLRVQLARH
PA genotype 4   ERRRKRNRDAARRRRREQTYYVDKLHEACEELQRANEHLRKEIRDLRTESTSLRVQLACH
PA genotype 1   ERRRKRNRDAARRRRRKQTDYVDKLHEACEELQRANEHLRKEIRDLRTECTSLRVQLARH
PA genotype 3   ERRRKRNRDAARRRRREQTYYVDKLHEACEELQRANEHLRKEIRDLRTECTSLRVQLARH
PA genotype 5   ERRRKRNRDAARRRRREQTYYVDKLHEACEELQRANEHLRKEIRDLRTECTSLRVQLARH
Rispens         ERRRKRNRDASRRRRREQTDYVDKLHEACEELQRANEHLRKEIRDLRTECTSLRVQLACH
                **********S***** ** ************************** ** ****A*** *

PA genotype 2   EPVCPMAVPLTVTLGLLTTPHDPVPEPPICTPQPPSPDEPNAPHCSGSQPPICTPAPPDT
PA genotype 4   EPVCPMAVPLTVTLGLITTPHDPVPEPPICTPPPPSPDEPNAPHCSGSQPPICTPPPPDT
PA genotype 1   EPVCPMAVPLTVTLGLLTTPHDPVPEPPICTPQPPSPDEPNAPHCSGSQPPICTPAPPDA
PA genotype 3   EPVCPMAVPLTVTLGLLTTPHDPVPEPPICTPPPPSPDEPNAPHCSGSQPPICTPPPPDT
PA genotype 5   EPVCPMAVPLTVTLGLLTTPHDPVPEPPICTPPPPSPDEPNAPHCSGSQPPICTPPPPDT
Rispens         EPVCPMAVPLTVTLGLLTTPHDPVPEPPICTPPPPSPDEPNAPHCSGSQPPICTPPPPDT
                **************** *A**N********** ********************** ***

PA genotype 2   EELCAQLCSTPP------------------------------------------------
PA genotype 4   EELCAQLCSTPP------------------------------------------------
PA genotype 1   EELCAQLCSTPP------------------------------------------------
PA genotype 3   EELCAQLCSTPS------------------------------------------------
PA genotype 5   EELCAQLCSTPP------------------------------------------------
```

```
Rispens         EELCAQLCSTPPPISTPHIIYAPGPSPLQPPICTPPPPDAEELCAQLCSTPPPPISTPHI
                ***********



PA genotype 2   -----------PPISTPHIIYAPGPSPLQPPICTPAPPDAEELCAQLCSTPPPPICTPHS
PA genotype 4   -----------PPISTPHIIYAPGPSPLQPPICTPAPPDAEELCAQLCSTPPPPICTPHS
PA genotype 1   -----------PPISTPHIIYAPGPSPLQPPICTPAPPDAEELCAQLCSTPPPPICTPHS
PA genotype 3   -----------PPISTPHIIYAPGPSPLQPPICTPAPPDAEELCAQLCSTPPPPICTPHS
PA genotype 5   -----------PPISTPHIIYAPGPSPLQPPICTPAPPDAEELCAQLCSTPPPPICTPHS
Rispens         FYAPGLCSTPPPPISTPHIIYAPGPSPLQPPICTPPPPDAEELCAQLCSTPPPPICTPHS
                           ************************P************************

PA genotype 2   LFCPPQPPSPEGIFPALCPVTEPCTPPSPGTVYAQLCPVGQVPLFTPSPPHPAPEPERLY
PA genotype 4   LFCPPQPPSPEGIFPALCPVTEPCAPPSPGTVYAQLCPVGQAPLFTPSPPHPAPEPERLY
PA genotype 1   LFCPPQPPSPEGIFPALCPVTEPCTPPSPGTVYAQLCPVGQAPLFTPSPPHPAPEPERLY
PA genotype 3   LFCPPQPPSPEGIFPALCPVTEPCTPPSPGTVYAQLCPVGQAPLFTPSPPHPTPEPERLY
PA genotype 5   LFCPPQPPSPEGIFPALCPVTEPCTPPSPGTVYAQLCPVGQAPLFTPSPPHPAPEPERLY
Rispens         LFCPPQPPSPEGIFPALCPVTEPCTPPSPGTVYAQLCPVGQAPLFTPSPPHPAPEPERLY
                ************************ **********P***** ********** *******

PA genotype 2   ARLTEDPEQDSLYSGQIYTQFPSDTQSTVWWFPGDGRP
PA genotype 4   ARLTEDPEQDSLYSGQIYIQFPSDTQSTVWWFPGDGRP
PA genotype 1   ARLTEDPEQDSLYSGQIYIQFPSDTQSTVWWFPGDGRP
PA genotype 3   ARLTEDPEQDSLYSGQIYIQFPSDTQSTVWWFPGDGRP
PA genotype 5   ARLTEDPEQDSLYSGQIYIQFPSDTQSTVWWFPGDGRP
Rispens         ARLTEDPEQDSLYSGQIYIQFPSDIQSTVWWFPGDGRP
                ****************** ***** *************
```

Residues in blue indicate the fourteen non-synonymous amino acid mutations discriminating the five PA genotypes. Red nucleotides indicate additional previously recorded residue differences among the ten strains with fully sequenced genomes. Gaps among asterisks indicate residue differences between PA genotypes and Rispens vaccine strain.

MARKER NO. 4: proximal region of ICP4 gene.

```
PA genotype 3 AAACCCCATTTTCGTGCAGCTCGGTTAGAATATGTCTCGCCGCAGAGAAAGTCTGGCCTC
PA genotype 1 AAACCCCATTTTCGTGCAGCTCGGTTAGAATATGTCTCGCCGCAGAGAAAGTCTGGCCTC
PA genotype 2 AAACCCCATTTTCGTGCAGCTCGGTTAGAATATGTCTCGCCGCAGAGAAAGTCTGGCCTC
              ************************************************************

PA genotype 3 ACGTGTTTAACTGCAGCAAAATAAAAGAGGGGAGTAGTAGGAACGCAAGATACGTCTTCC
PA genotype 1 ACGTGTTTAACTGCAGCAAAATAAAAGAGGGGAGTAGTAGGAACGCAAGATACGTCTTCC
PA genotype 2 ACGTGTTTAACTGCAGCAAAATAAAAGAGGGGAGTAGTAGGAACGCAAGATACGTCTTCC
              ****************T*******************************************

PA genotype 3 AGGCGCGTTTCGACTGTATACCGGACATTGCCGGTACAACATAGATTGATGGTACTGTCC
PA genotype 1 AGGCGCGTTTCGACTGTATACCGGACATTGCCGGTACAACATAGATTGATGGTACTGTCC
PA genotype 2 AGGCGCGTTTCGACTGTATACCGGACATTGCCGGTACAACATAGATTGATGGTACTGTCC
              ************************************************************

PA genotype 3 TCTATCCCAATTTCACAGTAATAATACTGTTCTAGATCCATGAGAGTTAATGGGCCGACA
PA genotype 1 TCTATCCCAGTTTCACAGTAATAATACTGTTCTAGATCCATGAGAGTTAATGGGCCGACA
PA genotype 2 TCTATCCCAATTTCACAGTAATAATACTGTTCTAGATCCATGAGAGTTAATGGGCCGACA
              ****C**** *****************************G********************
```

```
PA genotype 3 ACATCCCGCGAGGTTATAATAGTGGTCCCGAAATCGAGAAGGCCCGGCCAGCGGACACAG
PA genotype 1 ACATCCCGCGAGGTTATAATAGTGGTCCCGAAATCGAGAAGGCCGGGCCAGCGGACACAG
PA genotype 2 ACATCCCGCGAGGTTATAATAGTGGTCCCGAAATCGAGAAGGCCGGGCCAGCGGACACAG
              ************************************S******T ***************

PA genotype 3 CACTGACTACGGGGTGAAATTATACATTTCATGTAACGTGTCGCTTCTTCAATGATACAT
PA genotype 1 CACTGACTACGGGGTGAAATTATACATTTCATGTAACGTGTCGCTTCTTCAATGATACAT
PA genotype 2 CACTGACTACGGGGTGAAATTATACATTTCATGTAACGTGTCGCTTCTTCAATGATACAT
              ************************************************************

PA genotype 3 GGACCGTTTTGTAGTCTATATCGATCTTCTACCGTATCCATGATTATAAGTTTCTTTTTA
PA genotype 1 GGACCGTTTGGCAGTCTATATCGATCTTCTACCGTATCCATGATTATAAGTTTCTTTTTA
PA genotype 2 GGACCGTTTGGTAGTCTATATCGATCTTCTACCGTATCCATGATTATAAGTTTCTTTTTA
              ********* * ************************************************

PA genotype 3 CGTTTCATACATTGACGTTGTAGGTATTCCACAGCACCCTGATAGCCCAAATCCTCTGTT
PA genotype 1 CGTTTCATACATTGACGTTGTAGGTATTCCACAGCACCCTGATAGCCCAAATCCTCTGTT
PA genotype 2 CGTTTCATACATTGACGTTGTAGGTATTCCACAGCACCCTGATAGCCCAAATCCTCTGTT
              ************************************************************

PA genotype 3 GATAAAAACAGGACTCCCATCCGAGTCAGTGGAGATATATCGGGTGCTGCTTTCCATCGG
PA genotype 1 GATAAAAACAGGACTCCCATCCGAGTCAGTGGAGATATATCGGGTGCTGCTTTCCATCGG
PA genotype 2 GATAAAAACAGGACTCCCATCCGAGTCAGTGGAGATATATCGGGTGCTGCTTTCCATCGG
              ************************************************************

PA genotype 3 CCAGCCCACGCACTGCTCTTGGGTAAGCATAATCGGTTAGAAAATGCAGCAAGGAGGAAT
PA genotype 1 CCAGCCCACGCACTGCTCTTGGGTAAGCATAATCGGTTAGAAAATGCAGCAAGGAGGAAT
PA genotype 2 CCAGCCCACGCACTGCTCTTGGGTAAGCATAATCGGTTAGAAAATGCAGCAAGGAGGAAT
              *******************C****************************************

PA genotype 3 GATAATCCTCCTCGCTTATTATCAAACTTCAGCCCTCCCGGGGGAGTATCTGGAGCGGGT
PA genotype 1 GATAATCCTCCTCGCTTATTATCAAACTTCAGCCCTCCCGGGGGAGTATCTGGAGCGGGT
PA genotype 2 GATAATCCTCCTCGCTTATTATCAAACTTCAGCCCTCCCGGGGGAGTATCTGGAGCGGGT
              ******************************G*****************************

PA genotype 3 ACGCAAAACAGGTGTTCGCCTGGGAGGGGTGAGTATAAAACAACGATAGACACATCATCG
PA genotype 1 ACGCAAAACAGGTGTTCGCCTGGGAGGGGTGAGTATAAAACAACGATAGACACATCATCG
PA genotype 2 ACGCAAAACAGGTGTTCGCCTGGGAGGGGTGAGTATAAAACAACGATAGACACATCATCG
              ************************************************************

PA genotype 3 GGAGATTCTTGTTGGTATCGCATCCAAGCAGTCCTTGCTCTAAGCTTTGAAGTGGCTGTG
PA genotype 1 GGAGATTCTTGTTGGTATCGCATCCAAGCAGTCCTTGCTCTAAGCTTTGAAGTGGCTGTG
PA genotype 2 GGAGATTCTTGTTGGTATCGCATCCAAGCAGTCCTTGCTCTAAGCTTTGAAGTGGCTGTG
              *********************************************A**************

PA genotype 3 TGAAATGCCTCAGATGCAGCGCTAGTTATAATTTCGTTACTATTATATTTTCCAGGTAAC
PA genotype 1 TGAAATGCCTCAGATGCAGCGCTAGTTATAATTTCGTTACTATTATATTTTCCAGGTAAC
PA genotype 2 TGAAATGCCTCAGATGCAGCGCTAGTTATAATTTCGTTACTATTATATTTTCCAGGTAAC
              ************************************************************

PA genotype 3 GCATTTGCG
PA genotype 1 GCATTTGCG
PA genotype 2 GCATTTGCG
              *********
```

Nucleotides in blue indicate the four SNP sites discriminating the three PA genotypes. Red nucleotides indicate additional previously recorded SNP sites among the ten strains with fully sequenced genomes. Rispens vaccine sequence is identical to PA genotype no. 2. Accession numbers: PA1, MK084986; PA2, MK084987; PA3, MK084908.

MARKER NO. 5: distal region of ICP4 gene.

```
PA genotype 5 GAGGAGGATGTCACCCTGAATATCATTGCGTTGGACGGCTCGGCCGGACTTGGGAGGACG
PA genotype 2 GAGGAGGATGTCACCCTGAATATCATTGCGTTGGACGGCTCGGCCGGACTTGGGAGGACG
PA genotype 1 GAGGAGGATGTCACCCTGAATATCATTGCGTTGGACGGCTCGGCCGGACTTGGGAGGACG
PA genotype 6 GAGGAGGATGTCACCCTGAATATCATTGCGTTGGACGGCTCGGCCGGACTTGGGAGGACG
PA genotype 4 GAGGAGGATGTCACCCTGAATATCATTGCGTTGGACGGCTCGGCCGGACTTGGGAGGACG
PA genotype 3 GAGGAGGATGTCACCCTGAATATCATTGCGTTGGACGGCTCGGCCGGACTTGGGAGGACG
              ************************************************************

PA genotype 5 CCCACGTGGGCGTCCTCCTCCACGCCTGTTTGCGGTTTCTTGGGGTAAGTAGTCCTCATC
PA genotype 2 CCCACGTGGGCGTCCTCCCCCACGCCTGTTTGCGGTTTCTTGGGGTAAGTAGTCCTCATC
PA genotype 1 CCCACGTGGGCGTCCTCCCCCACGCCTGTTTGCGGTTTCTTGGGGTAAGTAGTCCTCATC
PA genotype 6 CCCACGTGGGCGTCCTCCTCCACGCCTGTTTGCGGTTTCTTGGGGTAAGTAGTCCTCATC
PA genotype 4 CCCACGTGGGCGTCCTCCTCCACGCCTGTTTGCGGTTTCTTGGGGTAAGTAGTCCTCATC
PA genotype 3 CCCACGTGGGCGTCCTCCCCCACGCCTGTTTGCGGTTTCTTGGGGTAAGTAGTCCTCATC
              **************T*** *****************************************

PA genotype 5 TTCCAGTTTACGCGTTCGGGGCGATGTTTTTCTCCCCTTAGACTGCGGTCCGGATCGCCC
PA genotype 2 TTCCAGTTTACGCGTTCGGGGCGATGTTTTTCTCCCCTTAGACTGCGGTCCGGATCGCCC
PA genotype 1 TTCCAGTTTACGCGTTCGGGGCGATGCTTTTCTCCCCTTAGACTGCGGTCCGGATCGCCC
PA genotype 6 TTCCAGTTTACGCGTTCGGGGCGATGTTTTTCTCCCCTTAGACTGCGGTCCGGATCGCCC
PA genotype 4 TTCCAGTTTACGCGTTCGGGGCGATGTTTTTCTCCCCTTAGACTGCGGTCCGGATCGCCC
PA genotype 3 TTCCAGTTTACGCGTTCGGGGCGATGTTTTTCTCCCCTTAGACTGCGGTCCGGATCGCCC
              *********G**************** *********************************

PA genotype 5 ACCACGATTACTACCTCTGCCCCGACGCGGGCGGCTCGAACAATCAGCCCGCGGTCTGGG
PA genotype 2 ACCACGATTACTACCTCTGCCCCGACGCGGGCGGCTCGAACAATCAGCCCGCGGCCTGGG
PA genotype 1 ACCACGATTACTACCTCTGCCCCGACGCGGGCGGCTCGAAGAATCAGCCCGCGGTCTGGG
PA genotype 6 ACCACGATTACTACCTCTGCCCCGACGCGGGCGGCTCGAACAATCAGCCCGCGGTCTGGG
PA genotype 4 ACCACGATTACTACCTCTGCCCCGACGCGGGCGGCTCGAAGAATCAGCCCGCGGTCTGGG
PA genotype 3 ACCACGATTACTACCTCTGCCCCGACGCGGGCGGCTCGAACAATCAGCCCGCGGTCTGGG
              **************************************** ************* *****

PA genotype 5 GGGCGACGGAGATGGTGACCTAGATGATGAAGGTGAAGACGGGACGATTAGAGAAGATGA
PA genotype 2 GGGCGACGGAGATGGTGACCTAGATGATGAAGGTGAAGACGGGACGATTAGAGAAGATGA
PA genotype 1 GGGCGACGGAGATGGTGACCTAGATGATGAAGGTGAAGACGGGACGATTAGAGAAGATGA
PA genotype 6 GGGCGACGGAGATGGTGACCTAGATGATGAAGGTGAAGACAGGACGATTAGAGAAGATGA
PA genotype 4 GGGCGACGGAGATGGTGACCTAGATGATGAAGGTGAAGACGGGACGATTAGAGAAGATGA
PA genotype 3 GGGCGACGGAGATGGTGACCTAGATGATGAAGGTGAAGACGGGACGATTAGAGAAGATGA
              ************************************G*G* *******************

PA genotype 5 TGACGGTGAGGATGATGAAGAGGAGGAAGATGAAGAGGATGGCGATCTGGAAACATGTCC
PA genotype 2 TGACGGTGAGGATGATGAAGAGGAGGAAGATGAAGAGGATGGCGATCTGGAAACATGTCC
PA genotype 1 TGACGGTGAGGATGATGAAGAGGAGGAAGATGAAGAGGATGGCGATCTGGAAACATGTCC
PA genotype 6 TGACGGTGAGGATGATGAAGAGGAGGAAGATGAAGAGGATGGCGATCTGGAAACATGTCC
PA genotype 4 TGACGGTGAGGATGATGAAGAGGAGGAAGATGAAGAGGATGGCGATCTGGAAACATGTCC
PA genotype 3 TGACGGTGAGGATGATGAAGAGGAGGAAGATGAAGAGGATGGCGATCTGGAAACATGTCC
              ************************************************************

PA genotype 5 GACGTTCCGGTGAGTAGTATTGGATGGGGGATTCCGGGGAGGGGATATAATAATGTGCTC
PA genotype 2 GACGTTCCGGTGAGTAGTATTGGATGGGGGATTCCGGGGAGGGGATATAATAATGTGCTC
PA genotype 1 GACGTTCCGGTGAGTAGTATTGGATGGGGGATTCCGGGGAGGGGATATAATAATGTGCTC
PA genotype 6 GACGTTCCGGTGAGTAGTATTGGATGGGGGATTCCGGGGAGGGGATATAATAATGTGCTC
PA genotype 4 GACGTTCCGGTGAGTAGTATTGGATGGGGGATTCCGGGGAGGGGATATAATAATGTGCTC
PA genotype 3 GACGTTCCGGTGAGTAGTATTGGATGGGGGATTCCGGGGAGGGGATATAATAATGTGCTC
              A***********************************************************

PA genotype 5 ATTTGACGGTGAAGTCTCAGGGCCGCGTGGTCTGGGGAACTTCGTGGAGATGAGGTTGTG
PA genotype 2 ATTTGACGGTGAAGTCTCAGGGCCGCGTGGTCTGGGGAACTTCGTGGAGATGAGGTTGTG
PA genotype 1 ATTTGACGGTGAAGTCTCAGGGCCGCGTGGTCTGGGGAACTTCGTGGAGATGAGGTTGTG
PA genotype 6 ATTTGACGGTGAAGTCTCAGGGCCGCGTGGTCTGGGGAACTTCGTGGAGATGAGGTTGTG
PA genotype 4 ATTTGACGGTGAAGTCTCAGGGCCGCGTGGTCTGGGGAACTTCGTGGAGATGAGGTTGTG
```

```
PA genotype 3 ATTTGACGGTGAAGTCTCAGGGCCGCGTGGTCTGGGGAACTTCGTGGAGATGAGGTTGTG
              ************************************************************

PA genotype 5 TG
PA genotype 2 TG
PA genotype 1 TG
PA genotype 6 TG
PA genotype 4 TG
PA genotype 3 TG
              **
```

Nucleotides in blue indicate the five SNP sites discriminating the six PA genotypes. Red nucleotides indicate additional previously recorded SNP sites among the ten strains with fully sequenced genomes. Rispens vaccine sequence is identical to PA genotype no. 6. Accession numbers: PA1, MK084989; PA2, MK084990; PA3, MK084991; PA4, MK084992; PA5, MK084993; PA6, MK084994.

MARKER NO. 6: region of pp38 gene.

```
PA genotype 2 CGAAGCAGAACACGAAGGGCTGACGGCGTCTTGGGTCGCCCCCGCTCCCCAGGGTGGAAA
Rispens       CGAAGCAGAACACGAAGGGCTGACGGCGTCTTGGGTCGCCCCCGCTCCCCAGGGTGGAAA
PA genotype 1 CGAAGCAGAACACGAAGGGCTGACGGCGTCTTGGGTCGCCCCCGCTCCCCAGGGTGGAAA
              ************************************************************

PA genotype 2 AGGGGCGGAGGGCCGCGCAGGGGTCGCCGACGAGGCAGGGCATGGGAAAACAGAAGCGGA
Rispens       AGGGGCGGAGGGCCGCGCAGGGGTCGCCGACGAGGCAGGGCATGGGAAAACAGAAGCGGA
PA genotype 1 AGGGGCGGAGGGCCGCGCAGGGGTCGCCGACGAGGCAGGGCATGGGAAAACAGAAGCGGA
              ************************************************************

PA genotype 2 ATGCGCCGAGGACGGCGAGAAATGCGGGGACGCCGAGATGAGCGCTTTGGATCGGGTCCA
Rispens       ATGCGCCGAGGACGGCGAGAAATGCGGGGACGCCGAGATGAGCGCTTTGGATCGGGTCCA
PA genotype 1 ATGCGCCGAGGACGGCGAGAAATGCGGGGACGCCGAGATGAGCGCTTTGGATCGGGTCCA
              ************************************************************

PA genotype 2 GAGGGACCGGTGGAGATTCAGTTCTCCGCCCCCTCACTCTGGAGTCACGGGGAAGGGGGC
Rispens       GAGGGACCGGTGGAGATTCAGTTCTCCGCCCCCTCACTCTGGAGTCACGGGGAAGGGGGC
PA genotype 1 GAGGGACCGGTGGAGATTCAGTTCTCCGCCCCCTCACTCTGGAGTCACGGGGAAGGGGGC
              ************************************************************

PA genotype 2 TATTCCAATAAAGGGTGATGGGAAGGCGATAGAATGCCAGGAGCTAACCGGAGAGGGAGA
Rispens       TATTCCAATAAAGGGTGATGGGAAGGCGATAGAATGCCAGGAGCTAACCGGAGAGGGAGA
PA genotype 1 TATTCCAATAAAGGGTGATGGGAAGGCGATAGAATGCCAGGAGCTAACCGGAGAGGGAGA
              ************************************************************

PA genotype 2 GTGGCTGTCACAGTGGGAGGAGCTACCGCCTGAGCCCCGGAGGTCAGGGAATGAACATCT
Rispens       GTGGCTGTCACGGTGGGGGGAGCTACCGCCTGAGCCCCGGAGGTCAGGGAATGAACATCT
PA genotype 1 GTGGCTGTCACAGTGGGGGGAGCTACCGCCTGAGCCCCGGAGGTCAGGGAATGAACATCT
              *********** ***** ******************************************

PA genotype 2 TGACGAAAGTCGGTATGCGAAACAAACCGAAAGGGGTAGCTCTACGGGGAAAGAAGAGGG
Rispens       TGACGAAAGTCGGTATGCGAAACAAACCGAAAGGGGTAGCTCTACGGGGAAAGAAGAGGG
PA genotype 1 TGACGAAAGTCGGTATGCGAAACAAACCGAAAGGGGTAGCTCTACGGGGAAAGAAGAGGG
              ************************************************************
```

```
PA genotype 2 AGATGGTATGAAGCAGATGGGGGAGCTTGCCCAGCAGTGCGAAGGAGGAACATATGCGGA
Rispens       AGATGGTATGAAGCAGATGGGGGAGCTTGCCCAGCAGTGCGAAGGAGGAACATATGCGGA
PA genotype 1 AGATGGTATGAAGCAGATGGGGGAGCTTGCCCAGCAGTGCGAAGGAGGAACATATGCGGA
              ************************************************************

PA genotype 2 CTTGCTTGTCGAAGCAGAGCAAGCTGTTGTACATTCCGTTCGCGCATTAATGCTGGCCGA
Rispens       CTTGCTTGTCGAAGCAGAGCAAGCTGTTGTACATTCCGTTCGCGCATTAATGCTGGCCGA
PA genotype 1 CTTGCTTGTCGAAGCAGAGCAAGCTGTTGTACATTCCGTTCGCGCATTAATGCTGGCCGA
              ************************************************************

PA genotype 2 AAGACAAAACCCAAATATATTGGGGGAGCATTTGAATAAAAAACGGGTTCTTGTACAACG
Rispens       AAGACAAAACCCAAATATATTGGGGGAGCATTTGAATAAAAAACGGGTTCTTGTACAACG
PA genotype 1 AAGACAAAACCCAAATATATTGGGGGAGCATTTGAATAAAAAACGGGTTCTTGTACAACG
              ************************************************************

PA genotype 2 ACCCCGTACTATTCTATCCGTGGAGTCAGAGAATGCAACAATGCGTTCTTATATGCTGGT
Rispens       ACCCCGTACTATTCTATCCGTGGAGTCAGAGAATGCAACAATGCGTTCTTATATGCTGGT
PA genotype 1 ACCCCGTACTATTCTATCCGTGGAGTCAGAGAATGCAACAATGCGTTCTTATATGCTGGT
              ************************************************************

PA genotype 2 TACATTGATCTGTTCTGCAAAATCATTATTACTAGGATCGTGCATGTCATTTTTCGCTGG
Rispens       TACATTGATCTGTTCTGCAAAATCATTATTACTAGGATCGTGCATGTCATTTTTCGCTGG
PA genotype 1 TACATTGATCTGTTCTGCAAAATCATTATTACTAGGATCGTGCATGTCATTTTTCGCTGG
              ************************************************************

PA genotype 2 TATGTTAGTCGGTAGAACGGCAGACGTAAAAACACCATTATGGGATACTGTATGTTTGTT
Rispens       TATGTTAGTCGGTAGAACGGCAGACGTAAAAACACCATTATGGGATACTGTATGTTTGTT
PA genotype 1 TATGTTAGTCGGTAGAACGGCAGACGTAAAAACACCATTATGGGATACTGTATGTTTGTT
              ************************************************************

PA genotype 2 AATGGCTTTCTGTGCAGGCATTGTCGTTGGGGGAGTGGATTCTG
Rispens       AATGGCTTTCTGTGCAGGCATTGTCGTTGGGGGAGTGGATTCTG
PA genotype 1 AATGGCTTTCTGTGCAGGCATTGTCGTTGGGGGAGTGGATTCTG
              ********************************************
```

Nucleotides in blue indicate the single SNP site discriminating the two PA genotypes. The other gap among asterisks indicates the SNP differentiating between wild type virus and attenuated vaccine strains (Rispens and strain 814). Accession numbers: PA1, MK084995; PA2, MK084996.

MARKER NO. 7: region of UL36

```
PA genotype 1 ACCGCCACTACCGTTACATCAGTCGTATCTATCACCATCTTTGCCCATTCATCTAGAATT
PA genotype 2 ACCGCCACTACCGTTACATCAGTCGTATCTATCACCATCTTTGCCCATTCATCTAGAATT
              ************************************************************

PA genotype 1 ACCTCAGGGTTATCCATAATTTGCCCAAAATATGGGTCGTGTTTTCGATCAAGCGAACAG
PA genotype 2 ACCTCAGGGTTATCCATAATTTGCCCAAAATATGGGTCGTGTTTTCGATCAAGCGAACAG
              ************************************************************

PA genotype 1 AGTCCAGACGTCGATCTATCTCTTAACACGTTCGCAGCTAGCATGGGTGCGTTCGATGTA
PA genotype 2 AGTCCAGACGTCGATTTATCTCTTAACACGTTCGCAGCTAGCATGGGTGCGTTCGATGTA
              *************** *******************************************
```

```
PA genotype 1 GTTCTTGAATATTTCCTGCAATTTGTAGTAGCTTGTCTGCTGTCAGTAGAATCTGTCATT
PA genotype 2 GTTCTTGAATATTTCCTGCAATTTGTAGTAGCTTGTCTGCTGTCAGTAGAATCTGTCATT
              ************************************************************


PA genotype 1 TTACACTCAATTTATATGATTGAAAATATGCGTCGAAACGACATAAGCTCCACTGCTGCC
PA genotype 2 TTACACTCAATTTATATGATTGAAAATATGCGTCGAAACGACATAAGCTCCACTGCTGCC
              ********************************************T***************

PA genotype 1 ATAAAGTATTTATACAAGTCATATACTGGGCTGAAGTTAAATTCGGATATTTGACGAGGC
PA genotype 2 ATAAAGTATTTATACAAGTCATATACTGGGCTGAAGTTAAATTCGGATATTTGACGAGGC
              ************************************************************


PA genotype 1 GC
PA genotype 2 GC
              **
```

Nucleotides in blue indicate the single SNP site discriminating the two PA genotypes. The red nucleotide indicates an additional previously recorded SNP site among the ten strains with fully sequenced genomes. Rispens vaccine sequence is identical to PA genotype no. 2. Accession numbers: PA1, MK084997; PA2, MK084998.

.

MARKER NO. 8: region of UL43

```
PA genotype 1     TGGTACTCGGGCCAACTTTATTTTCCGCATGTGCAGCAGCTTTGTCATGTTATACCTGCA
PA genotype 2     TGGTACTCGGGCCAACTTTATTTTCCGCATGTGCAGCAGCTTTGTCATGTTATACCTGCA
                  ********************************************G**********T****


PA genotype 1     TTAATATAAGGAATGCAAATAAGGGAATTAAACAATTAGCAGCTGCCTATATAGTGAAAT
PA genotype 2     TTAATATAAGGAATGCAAATAAGGGAATTAAACAATTAGCAGCTGCCTATGTAGTGAAAT
                  ************************************************** *********


PA genotype 1     CTATACTGGGATTTATCATAACTAGTTTACTTGTTTGTATATTAGTAGCGCTATCTTGAC
PA genotype 2     CTATACTGGGATTTATCATAACTAGTTTACTTGTTTGTATATTAGTAGCGCTATCTTGAC
                  ***********************************************************T

PA genotype 1     -CAAATCGTTGTTCACATCTTGGCCATATACGTATTGATCGTTGTTTCGAACCGCGAATA
PA genotype 2     -CAAATCGTTGTTCACATCTTGGCCATATACGTATTGATCGTTGTTTCGAACCGCGAATA
                  C**********************T************************************

PA genotype 1     AAACTTTCATACATACTAAACGATGGAGTTGTGTTTTATGAGCGTTGAAAACAAAGGTAC
PA genotype 2     AAACTTTCATACATACTAAACGATGGAGTTGTGTTTTATGAGCGTTGAAAACAAAGGTAC
                  ************************************************************

PA genotype 1     CATCGG
PA genotype 2     CATCGG
                  ******
```

Nucleotides in blue indicate the single SNP site discriminating the two PA genotypes. The red nucleotides indicate additional previously recorded SNP sites among the ten strains with fully sequenced genomes. Rispens vaccine sequence is identical to PA genotype no. 2. Accession numbers: PA1, MK084999; PA2, MK085000.

Accession numbers for the MDV-1 isolates utilized in Figure 6.

648A	AY362725
LMS	HQ858622
GA	M89471
RB-1B	AY243332
CU-2	AY362708
GX0101	JX844666.1
MD5	AF243438
MD11	AF493558.1
814	AF493551.1
CV1988 (Rispens)	AF493555.1
686	AY362727
AF	AF49380
AY	AY571784
Hmtib	ABA54944
BC	AY362707
584A	EU62706.5
X	AY362724
W	AY362723
U	AY362722
TK	AY362721
RL	AY362720
NEW	AY362719
N	AY362718
L	AY362717
567	AY362709
571	AY362710
573	AY362711
617A	AY362712
637	AY362713
549	AY362714
595	AY362715
643P	AY362716
JM	DQ534539
YA	HQ638156
WS03	HQ638152
MS57	EF523771
0095	AF493552.1

G2	AF493556.1
ATE	AY571784
C12_130	FJ436096.1
TN-N2	MH749325.1
3004	EU032468
4CRE	EF523772
02LAR	EF523773
FT158	EF523774
WOODLANDS	EF523775
MPF57	EF523771
BY	HM991861
GX060167	EU697887
GX070060	EU427303.1
GX070079	EU427304.1
TN-N1	HM749324.1
TN-N3	HM749326.1
J-1	HQ190957
YLO40920	DQ174459
LMEQ	DQ453117
0093CH	AF493550.1
0297CH	AF493553.1
0304CH	AF493554.1
LLY	HQ658621.1
LYC	HQ658627.1
LZY	HQ658609.1
LFY	HQ658615.1
LSY	HQ658626.1
LCGZ	HQ658612.1
LCD	HQ658611.1
LCZ	HQ658613.1
LHUAY	HQ658620.1
LQQHR	HQ658624.1
LSY2	HQ658625.1
LDH	HQ658614.1
LHC2	HQ658616.1
LHC3	HQ658617.1
LNMG	HQ658623.1
LHC4	HQ658618.1
LHC5	HQ658619.1
LCC	HQ658610.1
TOK_W2	AB638846
TOK_S1	AB638844
TOK_P1	AB638843
TOK_M2	AB638842
TOK_M1	AB638841
